# A Novel Ferroptosis-Related 4-Gene Prognostic Signature for Cholangiocarcinoma and Photodynamic Therapy

**DOI:** 10.3389/fonc.2021.747445

**Published:** 2021-10-12

**Authors:** Zi-jian Zhang, Yun-peng Huang, Xiao-xue Li, Zhong-tao Liu, Kai Liu, Xiao-feng Deng, Li Xiong, Heng Zou, Yu Wen

**Affiliations:** Department of General Surgery, The Second Xiangya Hospital, Central South University, Changsha, China

**Keywords:** ferroptosis, photodynamic therapy, cholangiocarcinoma, prognosis, ROS - reactive oxygen species

## Abstract

Cholangiocarcinoma is the second most common malignant tumor in the hepatobiliary system. Compared with data on hepatocellular carcinoma, fewer public data and prognostic-related studies on cholangiocarcinoma are available, and effective prognostic prediction methods for cholangiocarcinoma are lacking. In recent years, ferroptosis has become an important subject of tumor research. Some studies have indicated that ferroptosis plays an important role in hepatobiliary cancers. However, the prediction of the prognostic effect of ferroptosis in patients with cholangiocarcinoma has not been reported. In addition, many reports have described the ability of photodynamic therapy (PDT), a potential therapy for cholangiocarcinoma, to regulate ferroptosis by generating reactive oxygen species (ROS). By constructing ferroptosis scores, the prognoses of patients with cholangiocarcinoma can be effectively predicted, and potential gene targets can be discovered to further enhance the efficacy of PDT. In this study, gene expression profiles and clinical information (TCGA, E-MTAB-6389, and GSE107943) of patients with cholangiocarcinoma were collected and divided into training sets and validation sets. Then, a model of the ferroptosis gene signature was constructed using least absolute shrinkage and selection operator (LASSO)-penalized Cox regression analysis. Furthermore, through the analysis of RNA-seq data after PDT treatment of cholangiocarcinoma, PDT-sensitive genes were obtained and verified by immunohistochemistry staining and Western blot. The results of this study provide new insight for predicting the prognosis of cholangiocarcinoma and screening target genes that enhance the efficacy of PDT.

## Introduction

Cholangiocarcinoma (CCA) is an invasive epithelial malignant tumor derived from the bile ducts, and its incidence has shown a significant upward trend worldwide (1). Anatomically, it can be divided into intrahepatic CCA and extrahepatic CCA (2). The former is the most common primary bile duct cancer, and the latter is the second most common intrahepatic cancer (3). The clinical symptoms of CCA are mostly locally advanced or metastatic tumors, with insidious onset, high invasiveness, invasion into tissues around the liver, and lymph node and distant metastases. Therefore, most patients are in the advanced stage when they are first diagnosed. For resectable CCA, the significance of R0 resection is indisputable, but due to the complex anatomical structures of the hilum and intrahepatic bile duct and other characteristics, most patients have missed the opportunity for radical surgery once diagnosed (4). There are many reports that the R0 resection rate of CCA is <30% (5). Even in patients who conditionally undergo radical surgery, the postoperative recurrence rate is still 40%–80%, and the 5-year survival rate is only 20%–40% (6). Therefore, the early molecular diagnosis and prognostic judgment of cholangiocarcinoma are of great significance for improving the survival status of patients with CCA.

In recent years, many potential targets of diagnosis or treatment for CCA have been discovered. Ferroptosis is a recently discovered type of cell death that manifests as iron-dependent nonapoptotic cell death with reactive oxygen species (ROS) accumulation (7). Iron overload and ROS generation lead to lipid peroxidase in cell membranes, resulting in abnormal membrane shrinkage, fragmentation, cristae reduction, or the disappearance of mitochondria, which promotes cell death (8). Viswanathan et al. found that the resistance status of a variety of tumor treatments is closely related to the lipid peroxidase pathway and concluded that ferroptosis is a new method to overcome cancer treatment resistance (9). At present, the use of ferroptosis induction to reverse therapy resistance has been reported in colorectal cancer (10), ovarian cancer (11), and lung cancer (12), but there are very few prognostic studies in CCA. Therefore, it is necessary to explore the potential application value of ferroptosis in CCA.

Photodynamic therapy (PDT) is a new type of tumor treatment method that mainly produces ROS to kill cancer cells (13). The application of PDT to patients with advanced CCA through choledochoscopy is a very promising method for minimally invasive diagnosis and treatment. Because PDT does not have cross-resistance with chemotherapy, in 2014, the NCCN guidelines pointed out that endoscopic PDT through the biliary tract is an emerging palliative treatment for unresectable extrahepatic CCA (14). PDT combined with stent therapy achieves a better prognosis than biliary stent therapy alone (15). Studies have confirmed that biliary stent combined with PDT technology through endoscopic retrograde cholangiopancreatography can achieve better overall survival (OS) (14.2 *vs.* 9.8 months, p = 0.003) (16). Moreover, PDT can induce the overproduction of ROS and lipid peroxides (17). Therefore, some nano-materials have applied PDT to induce ferroptosis to treat tumors. However, in our recent study, it was found that PDT alone can upregulate the expression of the cystine transporter-XCT, which is not conducive to the occurrence of ferroptosis. These results reveal that the effect of PDT on CCA and ferroptosis is still uncertain, and it is necessary to propose potential molecules involved in PDT-mediated ferroptosis to truly integrate and apply this emerging therapy and understand its mechanisms.

## Materials and Methods

### Data Acquisition and Preprocessing

We downloaded three sets of RNA expression data with patient prognostic information from three platforms: the TCGA-CHOL data set (https://www.tcga.org), the GSE107943 data set from the Gene Expression Omnibus (GEO) database (https://www.ncbi.nlm.nih.gov/geo/), and the E-MTAB-6389 data set from the European Bioinformatics Institute (EMBL-EBI) database (https://www.ebi.ac.uk/). These data sets contain the following information: expression profiles obtained by array or high-throughput sequencing, patient age, histological type, and overall survival (OS). After screening, 36, 30, and 78 tumor tissue samples were obtained from the TCGA-CHOL, GSE107943, and E-MTAB-6389 data sets, respectively. According to the sample size, the E-MTAB-6389 data set was used as the training set, and the TCGA-CHOL and GSE107943 data sets were used as the validation sets. The microarray platform of the GSE107943 data set is GPL18573, and the microarray platform of the E-MTAB-6389 data set is GPL17585. According to the annotation file provided by the platform, we used the limma package in R software to exclude coding genes with missing values, annotated the RNA expression data individually according to the gene name, and then used the log2 function to preprocess the gene expression values. For a gene containing multiple probes, the median was used to represent its expression value.

### Analysis of the Ferroptosis-Related Weighted Coexpression Gene Network Based on Patient Data

FerrDb is the first specialized ferroptosis regulatory gene and disease database that summarizes the possible ferroptosis marker genes that can upregulate or downregulate ferroptosis. We extracted and merged all types of ferroptosis genes in this database and ultimately obtained 275 ferroptosis genes. Then, through a comparison of the TCGA-CHOL, GSE107943, and E-MTAB-6389 data sets, 248 common ferroptosis-related genes (FRGs) were obtained. After calculating the Z-score of the clinical information of the E-MTAB-6389 data set, we used the “WGCNA” package in R software (18) to perform weighted gene coexpression network analysis of the FRGs in E-MTAB-6389. The specific process of WGCNA included cluster analysis of the FRGs based on their expression and calculation of the association between each clustering module and the clinical phenotype. In this study, the hub correlation threshold in WGCNA was set to 0.9, the minimum number of modules was set to 20, the threshold of module merging was set to 0.25, the sensitivity was set to 2, and the rest of the parameters were the default values. A clustering dendrogram was used to display the results of gene merging and classification. Finally, Pearson correlation analysis was used to evaluate the correlations between different clustering modules and clinical features.

### Fer-Score Model Construction

The least absolute shrinkage and selection operator (LASSO) method is a compression estimation method that functions by shrinking the variable set (order reduction) (19). By constructing a penalty function, it can compress the coefficients of variables and make some regression coefficients become 0, thereby achieving the purpose of variable selection. Using the glmnet package in R software, we applied the LASSO Cox regression method to construct the Fer-score model of the FRGs with the best weighted coefficients. Furthermore, we drew the CV curve, cross-validated the penalty coefficient λ through the cross-validation method, and selected the λ value with the best cross-validation error. According to the best model parameters, the model was refitted with all the data. Finally, the Fer-score model that we obtained can be expressed concisely as follows: Fer-score = ∑Exp − Genei*βi. We also used univariate Cox regression analysis to determine the correlation between the Fer-score of a gene and patient prognoses in the E-MTAB-6389 training set.

### Fer-Score Model Verification

First, internal verification of the constructed Fer-score model was carried out. The Fer-score of each sample in the E-MTAB-6389 training data set was calculated using the ∑Exp − Genei*βi formula, and the sample score, distribution, and expression value of each gene were plotted. Furthermore, we used the “survival,” “KMsurv,” and “survivalROC” packages in R to perform Kaplan–Meier (KM) survival analysis and drew receiver operating characteristic (ROC) curves. Second, the TCGA-CHOL and GSE107943 validation data sets were used to verify the Fer-score model externally. According to the cutoff values in TCGA-CHOL and GSE107943, CCA patients were divided into high- and low-Fer-score groups to verify the robustness of the model. KM analysis of OS was used to test the distinguishing effect between CCA patients with high scores and those with low scores. Finally, ROC curves were used to evaluate the accuracy and predictive ability of the Fer-score.

### Gene Sets and Related Network Analysis

We first used gene set enrichment analysis (GSEA) to determine the potential signaling pathways associated with the genes in the Fer-score model (20). We used the “Kyoto Encyclopedia of Genes and Genomes” (KEGG) as the pathway template by downloading the c2.cp.kegg.v7.4.symbols.gmt and grouped the Fer-scores for enrichment analysis. To further explain the relationships between the pathways associated with the FRGs in CCA and patient prognoses, gene expression variation analysis (GEVA) was used to obtain the pathway score matrix (21). Then, the KM survival curve of the pathway was calculated with the “KMsurv” package.

### Immunohistochemistry

First, tissues were embedded and sliced. The slides were then placed in a series of xylene, xylene, 100% alcohol, 100% alcohol, 95% alcohol, 90% alcohol, 80% alcohol, and 70% alcohol for dewaxing. Antigen retrieval was performed, endogenous catalase was removed, and the antigen site was exposed. Serum was added to block some nonspecific sites. The serum around the back and front tissues of the slide was dried with absorbent paper. After adding the primary antibody, the cells were stored overnight in a refrigerator at 4°C. The secondary antibody was added, and the sample was placed in a 37°C incubator for half an hour. The film was removed from the incubator and washed with phosphate-buffered saline (PBS) three times, and a developer was added. The developed tissue was soaked in hematoxylin and dye for 30 s. The glass slide was placed into a series of 70% alcohol, 80% alcohol, 90% alcohol, 95% alcohol, 100% alcohol, 100% alcohol, xylene, and xylene for dehydration. Finally, neutral gum was placed on the side of the tissue, and the sample was covered with a cover slip.

### Cell Culture and Cell Viability for PDT

CCA and bile duct epithelial cell lines HUCCT-1 and RBE were purchased from the Cell Resource Centre of Shanghai Institutes for Biological Sciences and maintained in RPMI-1640 medium supplemented with 10% fetal bovine serum. Cell lines were routinely cultured at 37°C, with 21% O_2_ and 5% CO_2_ and they were tested negative for mycoplasma before any drug treatments were conducted.

HUCCT-1 and RBE cells were plated onto six-well plates for 24 h. After washing twice with PBS, the cells were incubated with 0–0.9 mM sinoporphyrin sodium (DVDMS) in 2 ml of medium containing 10% fetal bovine serum (FBS). After incubating in DVDMS for 4 h, we used a 630-nm wavelength laser to irradiate the six-well plate until the total energy density reached 10 J/cm^2^.

HUCCT-1 and RBE cells were seeded on 96-well plates at a density of 1.5 × 10^4^ cells/ml and cultured 24 h prior to drug treatment. Cell viability was determined using the Celltiter Blue^®^ reagent (Promega, G8082, United States). A 37°C water bath was used to thaw the Celltiter Blue^®^ reagent and bring it to ambient temperature. Assay plates were removed from the 37°C incubator, and 20 µl/well of Celltiter Blue^®^ Reagent was added. The plates were shaken for 10 s. Cells were incubated in standard cell culture conditions for 2 h. The plates were shaken for 10 s, and the fluorescence intensity was recorded at 560/590 nm.

### Colony Formation Experiment

HUCCT-1 and RBE cells were seeded on a six-well plate at a density of 1,000 cells/well. After 72 h, the cholangiocarcinoma cells were subjected to PDT treatment. On the 10th day, the medium was removed from the wells, the cells were washed twice with PBS, fixed in a 4% paraformaldehyde (PFA) incubator for 10 min, stained with a crystal violet solution for 10 min, and washed twice with PBS. Photos of the colonies in the wells were taken using a digital camera (iPhone X).

### Analysis of the ROS Levels

Briefly, cells that had undergone different drug treatments were harvested and resuspended in 1× PBS. To measure the intracellular ROS levels, cells were incubated with 10 μM DCFH-DA (Sigma-Aldrich, St. Louis, MO, USA) in the dark for 30 min. The stained cells were collected and resuspended in 1× PBS and then subjected to flow cytometer (Beckman Coulter Epics Altra, Miami, FL, USA) analysis.

### Analysis of Glutathione and Lipid Peroxide Content

Lysates of snap-frozen cells were used to determine glutathione (GSH) and malonaldehyde (MDA) concentration with the following kits: GSH and GSSG Assay Kit (Beyotime Biotechnology, S0053, China) and Lipid Peroxidation MDA Assay Kit (Beyotime Biotechnology, S0131, China).

### Culture and PDT of Organoid

Samples for organoid culture were collected from five patients with CCA. Specimens of at least 1 cm^3^ were collected, 4–5 ml of a preheated human tissue digestion solution was added per gram of specimen, and the mixture was digested in a shaker at 37°C for 2 h, followed by washing and passing through a 70-μm nylon filter, and the collected cell suspension was centrifuged at 8°C and 300×*g* for 5 min. The supernatant was discarded, and the procedure was repeated three to five times. After counting the cells, the 1,000 cells were resuspended in 50 μl of Matrigel, and a drop of BME2 was added to the center of 12-well plates to seed the cells. After plating, the medium was changed every 2–3 days, on average. Primary organoids need to be passaged after 5–7 days of culture. The passage method is basically the same as that of the cell line, but it does not require the use of pancreatin. The detailed method is described in our previous literature (22). After the organoids were successfully extracted and cultured for 3–5 days, a special organoid medium containing 0.4 mM DVDMS was prepared. After 6 h, the six-well plate was irradiated with a laser at 630 nm until the total energy density reached 10 J/cm^2^. Celltiter Blue^®^ Reagent was used for viability detection as above.

### Microarray Screening of Differentially Expressed Genes After PDT

Total RNA was extracted with TRIzol and quantified on a NanoDrop 2000 (Thermo Scientific). RNA integrity was determined with an Agilent Bioanalyzer 2100 (Agilent Technologies). The Agilent Human lncRNA Microarray V6 (4*180K; Design ID, 084410) chip was used to detect the expression levels in RBE cells. After the samples were subjected to quality control, the data were standardized using the quantile algorithm. The standardized data were filtered, and in each group of samples used for comparisons, at least one group of 75% of the samples marked as detected probes was subjected to subsequent analyses. The p value and fold change value of the t-test were used to screen differential genes, and heatmaps and volcano plots were generated to visualize the differentially expressed genes. Image extraction and data acquisition of the original samples were completed by Oebiotech, and drawing and enrichment analysis were performed using the R script.

### Western Blot

Radioimmunoprecipitation assay (RIPA) buffer was used to lyse RBE and HUCCT-1 cells after DVDMS-PDT treatment to prepare protein samples. The samples were centrifuged at 16,000×*g* for 5 min at 4°C in a suitable centrifuge tube. The supernatant containing the cell extract was transferred to a new tube, 5× sodium dodecyl sulfate (SDS) loading buffer was added, and the mixture was denatured at 95°C for 5 min. Before electrophoresis, the bicinchoninic acid (BCA) method was used to analyze the concentration of protein. The electrophoretic conditions were 120 V for 50 min. After electrophoresis, a polyvinylidene fluoride (PVDF) membrane was used for protein transfer at 400 mA for 45 min. The PVDF membrane was blocked with nonfat milk containing TBST. SLC2A1 (Proteintech, 21829-1-AP)-, SLC2A6 (Abcam, ab119272)-, SLC7A5 (Proteintech, 13752-1-AP) and ZEB1 (Proteintech, 21544-1-AP)-corresponding polyclonal antibodies (all diluted 1:1,000) were used as primary antibodies. After overnight incubation, the cells were labeled with an horseradish peroxidase (HRP)-conjugated secondary antibody. Finally, the PVDF membrane was washed, developed with ECL solution, and photographed for archiving.

### Statistical Analysis

In this study, SPSS^©^ Statistics 25 and GraphPad Prism 8 were used for statistical analyses and drawing, and RNA microarray data were analyzed using R 3.6.2. All quantitative data are presented as the mean ± SEM and were obtained from at least three independent experiments. Analysis of data variance was performed using ANOVA. Levin’s variance equality test and two-sided t test were used for two independent samples. The log-rank test was used for survival analyses. The CompuSyn program was used to analyze combined effects. p < 0.05 indicated statistical significance. p < 0.05 is annotated with *, and p < 0.01 is annotated with **.

## Results

### Defining Clinical Features Related to FRGs

**Figure 1:**
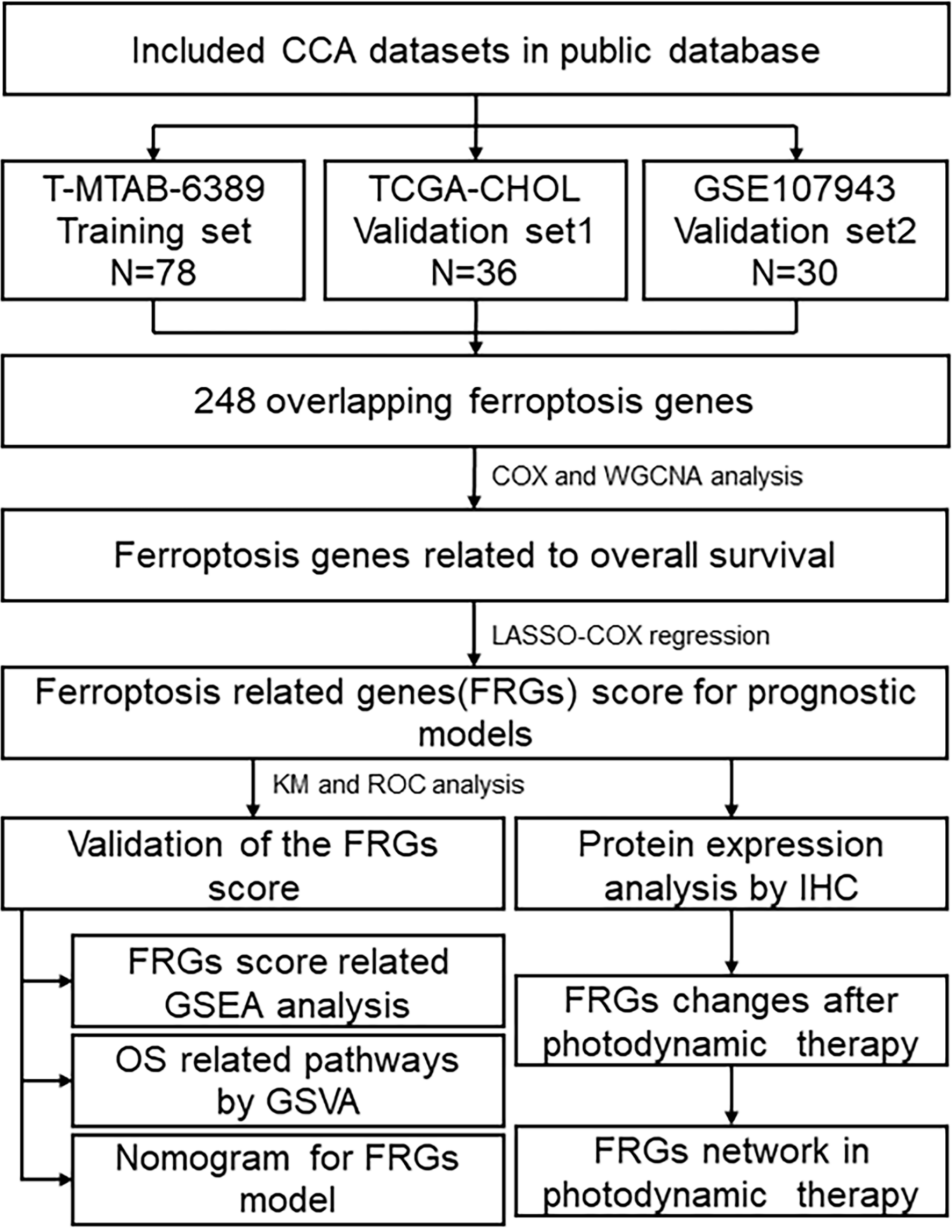
Flowchart describing the process used to identify and validate the Fer-score model of cholangiocarcinoma.

**Figure 2:**
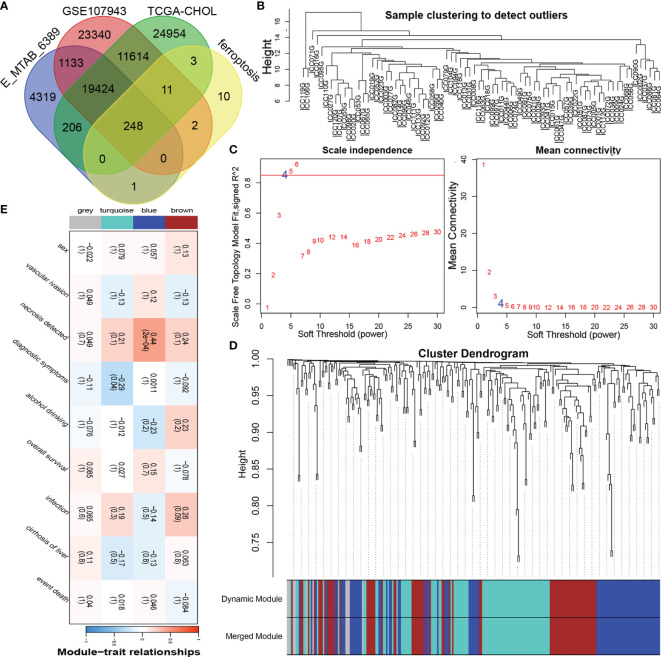
Construction of the coexpression modules of ferroptosis genes related to the clinical characteristics of cholangiocarcinoma. **(A)** Venn diagram of FRGs in cholangiocarcinoma. **(B)** Sample clustering of FRGs. **(C)** The soft threshold of the FRG module was defined by scale independence and mean connectivity. **(D)** Correlations between sample clustering and modules. **(E)** Relationships between the FRGs and clinical features.

The overall research process is shown in **Figure 1**. A total of 174 CCA patients from four independent cohorts in the E-MTAB, TCGA, and GEO databases and the Department of Hepatobiliary Surgery of Xiangya Second Hospital were included in our study. After obtaining FRGs from the FerrDb database and comparing the FRGs with those from existing studies, a total of 248 overlapping FRGs were selected (**Figure 2A** and **Supplementary Table S1**).

To obtain significantly representative genes and systematically classify these genes, we used WGCNA to construct a coexpression network and identify coexpression modules. Based on two evaluation perspectives—scale independence and mean connectivity—we determined the appropriate soft threshold capabilities from the scale-free topology model fit-R^2^ and thus divided the FRGs into 4 modules (**Figures 2B, C**). The correlations between each module and patient clinical characteristics (including sex, vascular invasion, necrosis, diagnostic symptoms, drinking, survival, infection, and cirrhosis) were calculated, as shown in **Figures 2D, E** and **Supplementary Figure 1**. The turquoise and blue modules were closely related to the clinical characteristics of CCA patients. The turquoise module contained 87 FRGs, and the blue module contained 80 FRGs (**Supplementary Table 1**); therefore, the information in these two main modules was selected for analysis.

### Construction of the Fer-Score Prognostic Model Based on FRGs Related to Clinical Characteristics

**Figure 3:**
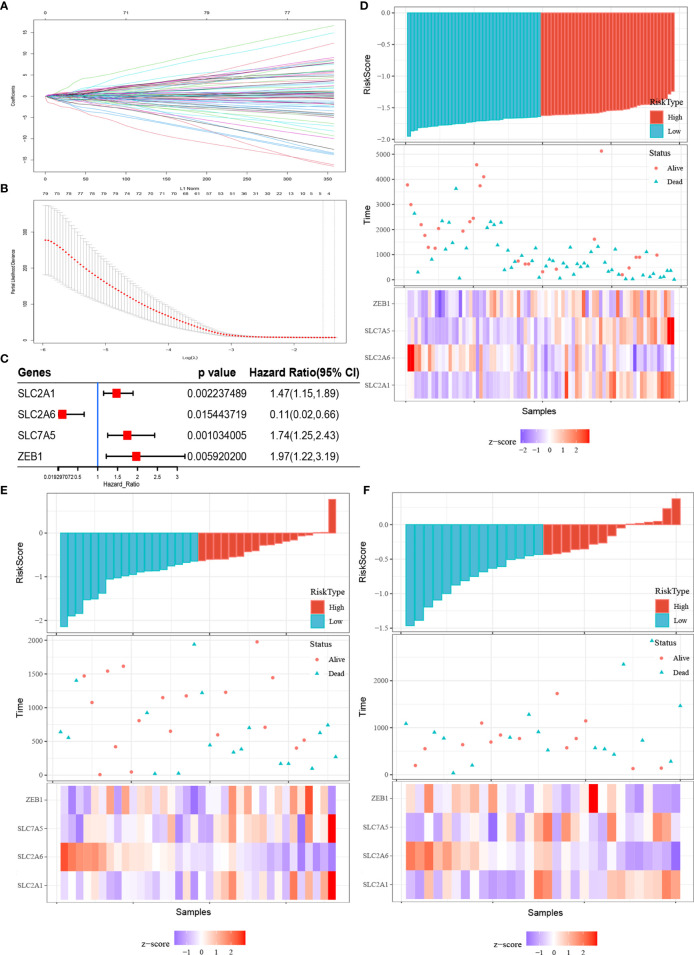
Fer-score model construction and visualization. **(A, B)** are the LASSO coefficient profiles and LASSO deviance profiles, respectively. **(C)** Forest plots of the Cox analysis of four genes screened by LASSO Cox. **(D–F)** Risk score (top panel), survival status (middle panel), and single gene expression level (bottom panel) of the E-MTAB-6389, TCGA-CHOL, and GSE107943 data sets.

To construct a simple and effective prognostic model, it was necessary to further reduce the dimensionality of the FRGs. LASSO Cox regression analysis is an effective machine learning method for feature selection when there are many variables and a limited number of samples. Through cross-validation (default number of times), four FRGs were incorporated into the model: SLC2A1, SLC2A6, SLC7A5, and ZEB1 (**Figures 3A, B**). The independent risk ratios of these four genes are shown as a forest diagram in **Figure 3C**. The risk ratios of SLC2A1, SLC7A5, and ZEB1 were 1.47, 1.74, and 1.97, respectively, while the risk ratio of SLC2A6 was 0.11 (p < 0.05). According to the expression values and correlation coefficients of these four genes, the prognostic risk score of each patient sample was calculated. **Figures 3D–F** show the total risk score (top panel), survival time (middle panel), and single gene expression level (bottom panel) of the E-MTAB-6389, TCGA-CHOL, and GSE107943 data sets. According to the Fer-score of each patient shown in **Figures 3D–F**, the optimal cutoff value was used to divide patients into high- and low-risk groups.

### Prognostic Performance Verification of the Fer-Score for the Training Data Set and the Validation Data Sets

**Figure 4:**
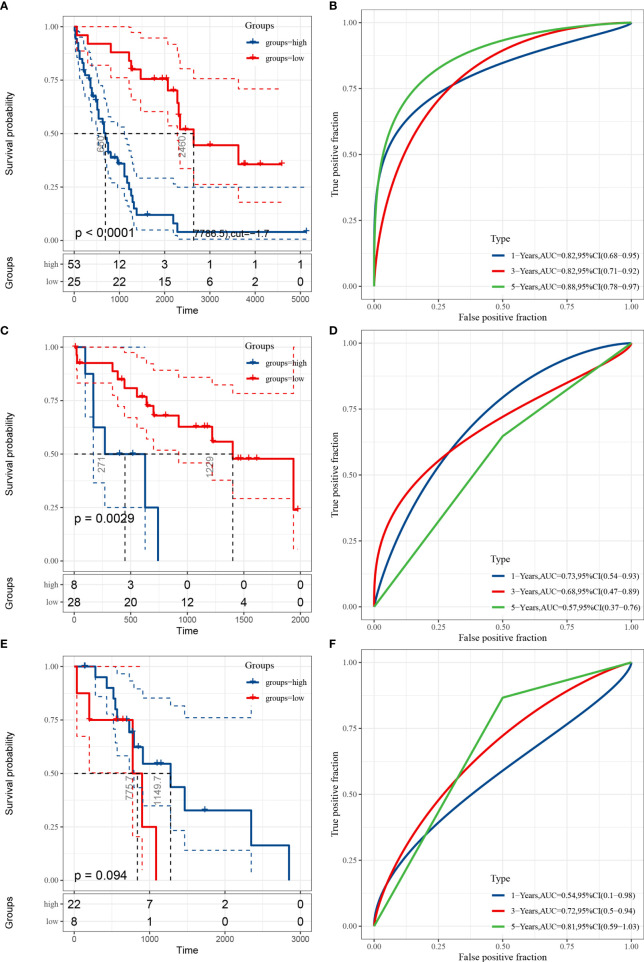
Evaluation and validation of the prognostic performance of the Fer-score in three independent cohorts. **(A, C, E)** KM survival curves according to the Fer-score in the E-MTAB-6389, TCGA-CHOL, and GSE107943 data sets. **(B, D, F)** ROC curves at 1, 3, and 5 years according to the Fer-score in the E-MTAB-6389, TCGA-CHOL, and GSE107943 data sets.

To verify the prognostic performance of the Fer-score signature on different data platforms, E-MTAB-6389 was used as the training set, and TCGA-CHOL and GSE107943 were used as the validation data sets. **Figures 4A, C, E** show the KM survival curves of E-MTAB-6389, TCGA-CHOL, and GSE107943, respectively. Among these data sets, the survival outcomes of patients in E-MTAB-6389 and TCGA-CHOL were significantly different (log-rank p < 0.01). The p value of the KM curve of GSE107943 was 0.094, which may have been due to the small sample size. ROC curves were used to evaluate the sensitivity and specificity of the Fer-score model for the prognoses of patients. The results showed that the E-MTAB-6389 data set worked best, with areas under the curve (AUCs) of 0.82, 0.82, and 0.88 at 1, 3, and 5 years, respectively (**Figure 4B**). The AUCs at 1, 3, and 5 years in the TCGA-CHOL data set were 0.73, 0.68, and 0.57, respectively. However, since there were no patients with survival times exceeding 2,000 days in TCGA-CHOL, the 5-year AUC may not be accurate (**Figure 4D**). The AUCs at 1, 3, and 5 years in the GSE107943 data set were 0.54, 0.72, and 0.81, respectively (**Figure 4F**). In general, the AUCs of OS showed a good prognostic ability of the Fer-score. It is worth noting that compared with other potential prognostic indicators based on ROC curve analysis, this Fer-score has better predictive power and accuracy (**Supplementary Figures 2A–C**).

### Construction, Correction, and Single-Gene Survival Analysis of the Nomogram Based on the Fer-Score Model

**Figure 5:**
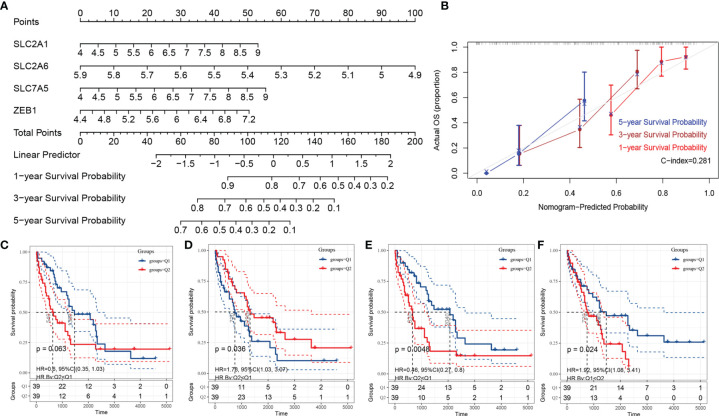
The nomogram of the Fer-score model and single-gene KM survival curve. **(A, B)** are the nomogram and calibration curve of the Fer-score model, respectively. **(B–F)** KM survival curves according to SLC2A1, SLC2A6, SLC7A5, and ZEB1 (Q1 is the low-risk group, and Q2 is the high-risk group).

To realize the application potential of the model, we integrated the risk score into a nomogram (**Figure 5A**). The calibration curve of the nomogram showed good agreement between the actual observed value and the predicted value (**Figure 5B**). In fact, the nomogram could also integrate risk scores with clinical characteristics to obtain better clinical utility. In addition, we performed survival analysis according to a single gene in the Fer-score. **Figures 5C–F** show the survival analysis according to the expression levels of SLC2A1, SLC2A6, SLC7A5, and ZEB1. Q1 is the low expression group, and Q2 is the high expression group. The survival of patients with high expression of SLC2A1, SLC7A5, and ZEB1 was worse than that of patients with low expression, while the opposite was true for SLC2A6. These results suggest that a single gene has a certain ability to distinguish patient survival times without the Fer-score.

### Fer-Score-Based Gene Set Enrichment and Mutation Analyses

**Figure 6:**
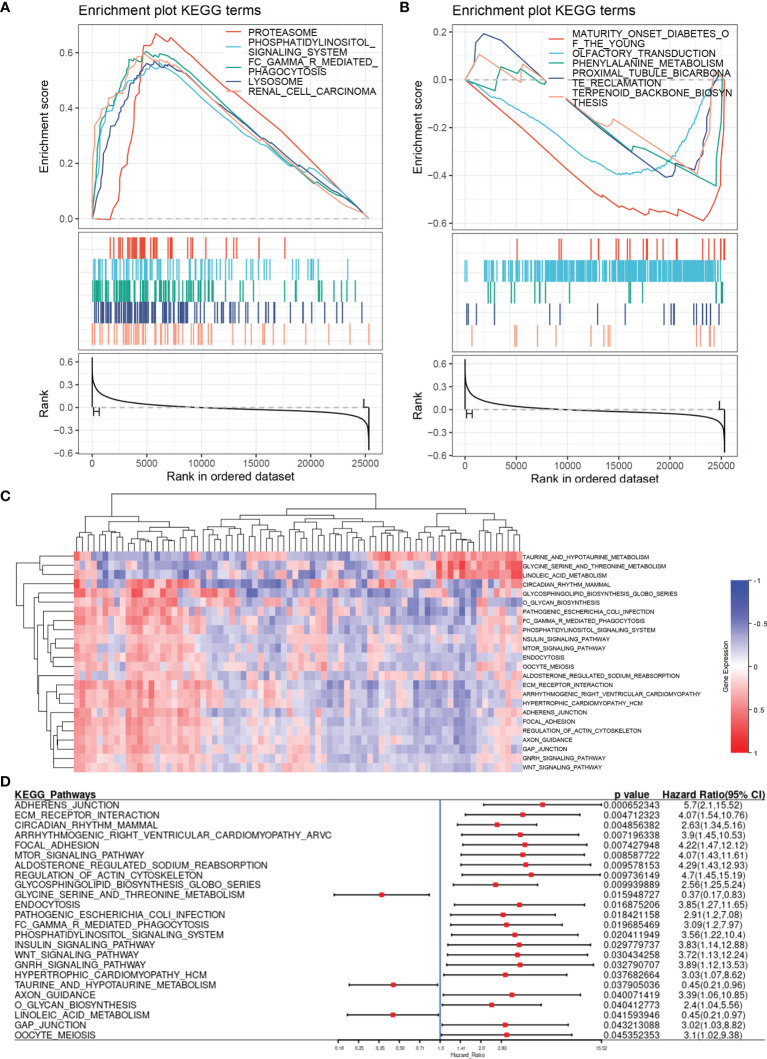
Pathway enrichment analysis based on the Fer-score. **(A, B)** are the enrichment pathways that are positively and negatively correlated with the Fer-score according to GSEA. **(C)** Heat map of the pathway variation matrix (by GSVA). **(D)** Forest diagram displaying the results of the Cox analysis of the pathway variation matrix.

Using the Fer-score as the basis for grouping, we performed GSEA on the FRGs to obtain the pathways that may participate in the regulation of the four genes included in the Fer-score. GSEA showed the 10 most important KEGG pathways that were most relevant to the Fer-score. Among them, renal cell carcinoma, lysosome-, FCγR-mediated phagocytosis, the phosphatidylinositol signaling system, and proteasome pathways were positively correlated, with normalized enrichment scores (NESs) of 1.8388997, 1.8042084, 1.7422546, 1.7368004 and 1.721969, respectively (**Figure 6A**). The tubule bicarbonate reclamation and terpenoid backbone biosynthesis pathways were negatively correlated, with NESs of −1.4151672, −1.3257339, −1.1133444, −0.9730135 and −0.9798353, respectively (**Figure 6B**).

Gene set variation analysis (GSVA) does not require grouping samples in advance and can calculate the enrichment score of a specific gene set in each sample. In other words, the GSVA algorithm transforms a single gene expression matrix into a specific gene set expression matrix (**Figure 6C**). We used KEGG gene sets to quantify the pathways and analyze the specific results of the aforementioned specific pathways and patient survival. By combining the results of GSEA and GSVA, we found that the FCγR-mediated phagocytosis and phosphatidylinositol signaling system pathways were closely related to the Fer-score signature (**Figure 6D**). The activation of these pathways is not conducive to patient survival.

### Verification of the Expression and Prognostic Value of the Fer-Score Signature Through Histopathology

**Figure 7:**
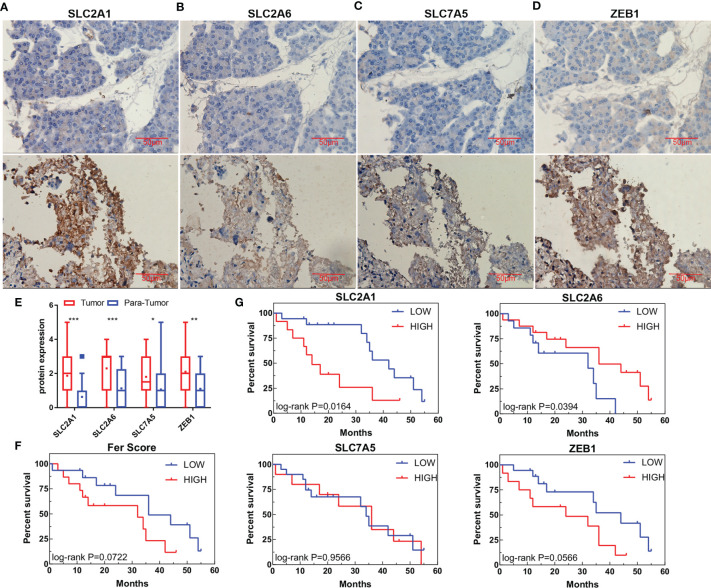
Protein expression and survival according to genes in the Fer-score model. **(A–D)** The expression of SLC2A1, SLC2A6, SLC7A5, and ZEB1 in adjacent tissues (top panel) and tumor tissues (bottom panel). **(E)** Statistical graphs of adjacent tissues and tumor expression. (Bar, 50 μm) **(F)** Survival curve based on the Fer-score model. **(G)** Survival curve according to the protein expression of the four genes. *p < 0.05, **p < 0.01, ***p < 0.001.

As the pathogenesis of CCA is complex, with significant regional and ethnic differences, it is necessary to revalidate the model for CCA patients in this region. By collecting tissue samples and follow-up data from 30 patients after surgery and determining the expression of SLC2A1, SLC2A6, SLC7A5, and ZEB1 in the samples, we analyzed the differences in protein expression and their impact on survival. The results showed that SLC2A1, SLC2A6, and SLC7A5 are mainly expressed on the cell membrane and may also be expressed in the cytoplasm when their expression is high, while ZEB1 is expressed in the nucleus and plasma (**Figures 7A–D**). Their expression in tumor tissue was higher than that in adjacent tissues (**Figure 7E**). After follow-up, the survival times of patients with high SLC2A1 and ZEB1 expression tended to be shorter (p = 0.0164 and 0.0566, respectively), while the survival times of patients with high SLC2A6 expression were prolonged (P=0.0394). These results are consistent with the gene expression results of public databases. The expression of SLC7A5 alone had no effect on survival (p = 0.9566). We further used the Fer-score model to calculate the total score of the immunohistochemistry (IHC) samples and found that the survival times of patients with high scores may also be short (p = 0.0722).

### PDT Effectively Kills CCA Cell Lines and Organoids and Induces Ferroptosis by Increasing ROS and MDA

**Figure 8:**
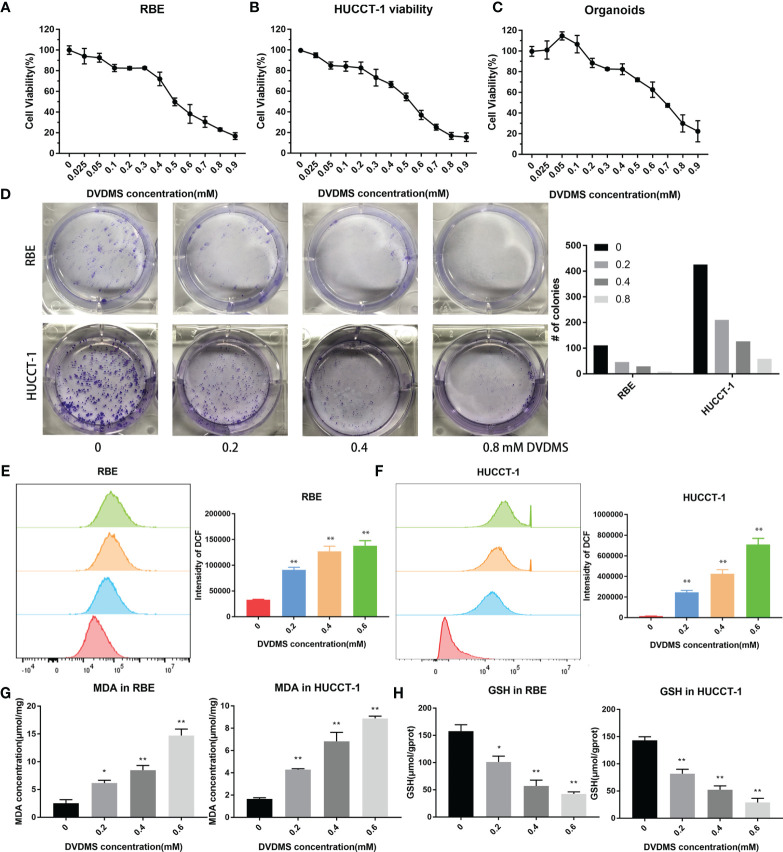
PDT inhibited the viability of CCA, increased the content of ROS and MDA, and decreased the concentration of GSH. **(A–C)** Celltiter blue was used to detect the viability changes of RBE, HUCCT-1, and organoids after different concentrations of DVDMS-PDT (0–0.9 mM, 10 J/cm^2^). **(D)** The images of colony formation and histograms show that different concentrations of DVDMS-PDT inhibited the proliferation in RBE and HUCCT-1. **(E, F)** Flow cytometry and histograms show that ROS levels upregulated in RBE and HUCCT-1 cells after DVDMS-PDT (0–0.6 mM, 10 J/cm^2^) treatment. **(G)** MDA increased and **(H)** GSH decreased after DVDMS-PDT (0–0.6 mM, 10 J/cm^2^) treatment, respectively. *p < 0.01, **p < 0.001.

We detected the inhibitory effect of DVDMS-mediated PDT (DVDMS-PDT) on RBE and HUCCT-1 cells. We found that DVDMS-PDT could reduce cell viability in a dose-dependent manner (**Figures 8A, B**). The same result was also observed after PDT treatment in the organoid model of CCA (**Figure 8C**). Colony formation experiments more intuitively confirmed the viability inhibition effect of DVDMS-PDT (0–0.8 mM, 10 J/cm^2^) on RBE and HUCCT-1 cells (**Figure 8D**). Based on this, the theoretical basis of the effect of DVDMS-PDT is to generate ROS. We used the ROS probe DCFH-DA to detect the levels of ROS when DVDMS-PDT was used in different concentrations (0–0.6 mM, 10 J/cm^2^) (**Figures 8E, F**). The results showed that, compared with the control group, the ROS levels of the DVDMS-PDT group were significantly increased in a dose-dependent manner.

To clarify whether DVDMS-PDT can induce ferroptosis in CCA cells, RBE and HUCCT-1 cells were treated with different concentrations of DVDMS (0–0.6M) for 48 h. MDA is an important metabolite of lipid peroxidation and biomarkers of ferroptosis in cells (23). Therefore, the content of MDA in CCA cells was evaluated by using MDA assay kit. As the concentration of DVDMS increases, the intracellular MDA levels in HUCCT-1 and RBE cells decrease (**Figure 8G**). GSH, which reduces ROS, is a product of glutathione peroxidase and can reflect the degree of ferroptosis. Compared with the control group, DVDMS-PDT inhibited the concentration of GSH (**Figure 8H**).

### Analysis of FRGs Regulated by PDT in the Treatment of CCA

**Figure 9:**
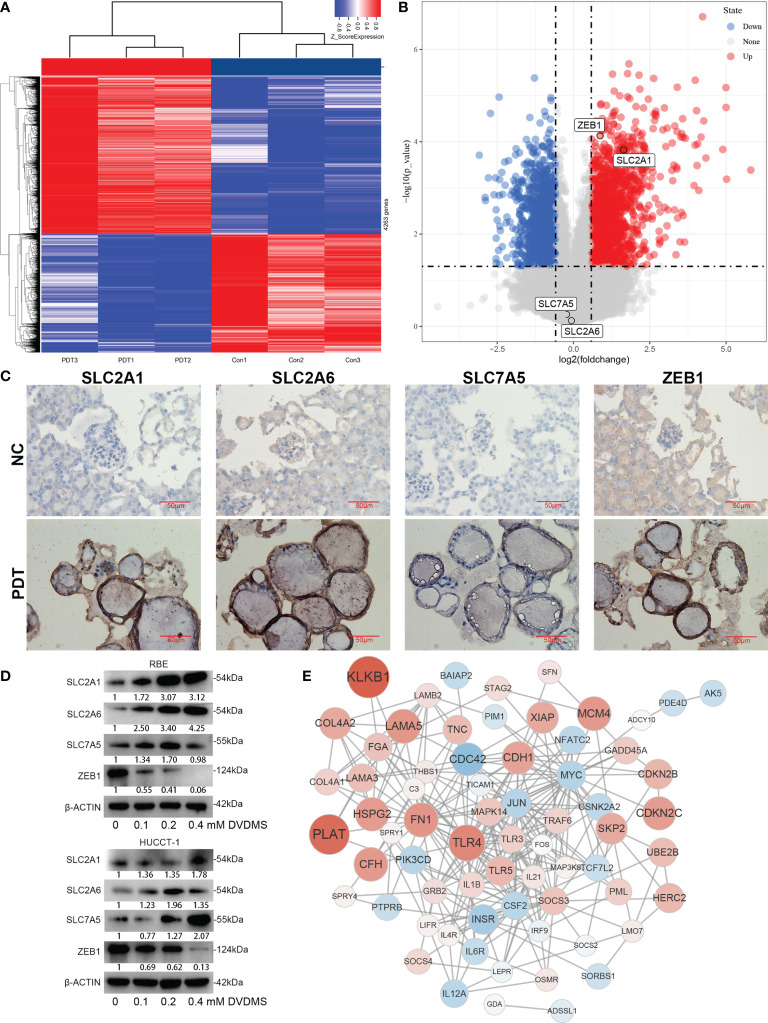
Expression changes and network analysis of Fer-score model genes in PDT. **(A, B)** The heatmap and volcano plot show the changes of genes after PDT treatment of RBE cells. Among them, red represents upregulation of gene expression, and blue represents downregulation of gene expression. **(C)** Protein expression changes in organoids of CCA after PDT treatment. **(D)** Protein expression changes in RBE and HUCCT-1 cells after PDT treatment (bar, 50 μm). **(E)** Construction of the gene network based on microarray data.

After DVDMS-PDT treatment, RBE cells were subjected to an RNA microarray. After removing duplicates, the results suggested that there were differences in the expression of 1,087 genes (**Figure 9A**), among which SLC2A1 was upregulated after treatment and ZEB1 was downregulated after treatment, while the expression of SLC2A6 and SLC7A5 was not significantly different (**Figure 9B**). In the organoid model treated with DVDMS-PDT, we also used IHC to detect changes in the expression of the above-mentioned proteins. The results showed that after DVDMS-PDT treatment, the expression of SLC2A1, SLC2A6, and SLC7A5 in the organoid model of CCA was also significantly higher than that in the untreated group (**Figure 9C**). Western blot analysis indicated that the expression of ZEB1 was significantly downregulated after treatment with different concentrations of PDT. In RBE and HUCCT-1 cells, the expression of SLC2A1, SLC2A6, and SLC7A5 was significantly increased, and the expression of ZEB1 was significantly decreased (**Figure 9D**). Finally, GSEA was constructed based on the Fer-score grouping, and the genes involved in the top 5 pathways according to their NESs were selected to draw a protein–protein interaction network (**Figure 9E**).

## Discussion

CCA is the most common malignant tumor of the biliary tract and the second most common malignant tumor of the liver, and it has high morbidity and mortality rates. Selective induction of the programmed cell death of cancer cells is one of the most effective treatment methods for malignant tumors (24), and most of these methods are directly related to the prognoses of patients (25). To date, many composite biomarkers have been reported to compensate for the deficiencies of clinicopathological characteristics and optimize their prognostic value in cancer. Previous studies have developed simple mRNA integration markers to predict the prognosis of CCA (26, 27), but these studies were externally verified in a small number of patients. Some evidence shows that ferroptosis plays a vital role in the occurrence and treatment of CCA (28). However, there are no studies on FRGs for the systematic analysis of the prognosis of CCA. This study first established a prognostic model with the E-MTAB-6389 training set that integrates four genes related to ferroptosis: SLC2A1, SLC2A6, SLC7A5, and ZEB1. Then, the constructed model was verified in the TCGA-CHOL and GSE107943 validation data sets.

SLC2A1 (29) and SLC2A6 (30) belong to the glucose transporter family and are important membrane proteins responsible for the stable supply of mammalian glucose. SLC7A5 plays a role in the composition of amino acid transporters in the absorption and output of cystine, glutamine, and other amino acids, thereby regulating glutathione synthesis, autophagy, and glutamine decomposition (31). Inhibition of the interaction between SLC7A5 and SLC3A2 prevents glutamine and leucine from activating mammalian rapamycin (mTOR) complex 1 (32). SLC7A5 is overexpressed in a variety of malignant tumors, and it is positively correlated with adverse clinical outcomes in CCA (33). ZEB1 is a classic epithelial–mesenchymal transition regulatory transcription factor. The complex arrangement of genes that mediate the transition from epithelial cells to mesenchymal cells has been proven to be related to the development and metastasis of CCA (34). Moreover, ZEB1 is an important regulator and potential prognostic factor in CCA (35).

In addition, after verifying the prognostic model, GSEA was used to determine the main pathways involved in CCA by comparing the high- and the low-risk groups in E-MTAB-6389. Although the mechanism of CCA is still unknown, we conducted an in-depth study on CCA based on the concept of ferroptosis. According to KEGG functional analysis, the FRGs were mainly enriched in renal cell carcinoma, lysosomes, FCγR-mediated phagocytosis, the phosphatidylinositol signaling system, proteasomes, maturity onset diabetes of the young, olfactory transduction, phenylalanine metabolism, proximal tubule bicarbonate reclamation, and the terpenoid backbone biosynthesis pathway. Then, the GSVA expression matrix showed the pathways related to the OS of CCA patients. Activation of the phosphatidylinositol signaling system pathway is related to a poor prognosis. This pathway is responsible for extracellular signaling molecules that stimulate the production of the secondary messengers inositol 1,4,5-trisphosphate (IP3) and diacylglycerol (DG) (36). IP3-mediated calcium regulation is closely related to endoplasmic reticulum stress (37). It can be speculated that the interaction between ferroptosis and endoplasmic reticulum stress in CCA may be mediated by this pathway. The activation of FCγR-mediated phagocytosis is also related to a short survival time in CCA patients. This pathway has been considered an important part of the innate immune response (38). The acquisition of lysosomal proteases and the release of ROS are also mediated by this pathway (39). Considering the process of ROS release and fusion with lysosomes, we believe that FCγR-mediated phagocytosis mediates the balance between macroautophagy and ferroptosis. These results suggest that administering inhibitors or activating these survival-related pathways may improve the prognoses of patients.

Due to the large differences in the etiology and ethnicity of patients in public databases, we collected samples from CAA patients in our hospital for verification. It has been proven that all four genes in the signature are upregulated in CCA, and the Fer-score, which is calculated based on protein expression, can distinguish the survival times of patients to a certain extent. Although the p value was 0.0722, we think that this was due to the small sample size. On this basis, we explored the effects of PDT on CCA cell lines and organoid models. Cell viability and colony formation experiments confirmed the growth inhibitory effect of PDT in cell lines and organoids, while the increase in the fluorescence intensity of DCF confirmed that PDT can induce ROS in CCA, which may lead to an increase in MDA and a decrease in GSH. Both MDA and GSH are important metabolic processes for ferroptosis, suggesting that PDT has the potential to induce ferroptosis. Finally, we used the microarray data of PDT for CCA cells to analyze the PDT-mediated ferroptosis regulation of the abovementioned key genes. SLC2A1 and ZEB1 were significantly differentially expressed. Furthermore, Western blot and IHC staining analysis showed that ZEB1 was significantly upregulated after PDT, while SLC2A1, SLC2A6, and SLC7A5 were downregulated. Marie-Nicole once reported that 7 days after PDT treatment for head and neck squamous cell carcinoma, ZEB1 in the exosomes of patients was downregulated to maintain the tumor cell epithelial phenotype and avoid metastasis (40). The results of our study show that direct stimulation with PDT can significantly inhibit the mesenchymal phenotype of cells within 6 h; thus, ZEB1 may be an important antitumor molecule for PDT. Regarding the effect of GLUT1/6 on PDT, some researchers believe that the expression of GLUT1 promotes the enrichment of dendritic chlorin in mitochondria, thereby enhancing the effect of PDT (41), while others believe that highly expressed GLUT1 is the main reason for the poor effect of glycosylated photosensitizers in 3D tumor spheres (42). Our research supports the view that GLUT1 antagonizes the PDT effect of DVDMS. There are few studies on SLC7A5 and PDT, but the use of erastin to antagonize the SLC7A11-enhancing effect of PDT has been reported (43). According to this study, SLC7A5 may also be a resistance factor in PDT-induced ferroptosis. Taken together, these results indicate that the above genes may be targets for PDT in treating CCA sensitivity or tolerance through ferroptosis. PDT combined with these targets is expected to further improve the prognosis of CCA.

This study has some limitations. First, the data used to establish the predictive model were obtained from public databases. Although the data were verified using patient and cell samples, the results at the protein level may be different from those in public databases. Second, there was significant heterogeneity among the patients included in this study. For example, the public database includes both intrahepatic and extrahepatic CCA patients, while our study included only postoperative patients with hilar CCA to ensure the comparability of the follow-up results. The use of different samples may be one of the important reasons for the significant differences in the predictive effects of the model. Third, the number of patients included in this study was limited, which could also have caused errors in the results.

Overall, we constructed a prognostic model related to ferroptosis. The model was verified in the training set and validation sets. In addition, a variety of gene function and pathway enrichment analyses showed that ferroptosis is related to the upstream pathways of endoplasmic reticulum stress and autophagy. The activation or inhibition of these pathways may affect the OS of patients. More importantly, the expression of some FRGs was significantly different after PDT treatment, suggesting that these genes may act as promoting or antagonizing factors in PDT ferroptosis therapy, but further experimental verification is needed.

## Data Availability Statement

The datasets E-MTAB-6389 for this study can be found in the European Bioinformatics Institute (EMBL-EBI) database (https://www.ebi.ac.uk/arrayexpress/experiments/E-MTAB-6389/), the TCGA-CHOL data set (https://portal.gdc.cancer.gov/), and the GSE107943 data set from the Gene Expression Omnibus (GEO) database (https://www.ncbi.nlm.nih.gov/geo/query/acc.cgi?acc=gse107943).

## Ethics Statement

The studies involving human participants were reviewed and approved by Medical Ethics Committee of the Second Xiangya Hospital of Central South University. The patients/participants provided their written informed consent to participate in this study.

## Author Contributions

Z-JZ designed and wrote the paper. HZ, X-XL, Z-TL, Y-PH, KL, LX, and X-FD edited the work. YW and HZ reviewed and revised the manuscript. All authors contributed to the article and approved the submitted version.

## Funding

This research was supported by Fundamental Research Funds for the Central Universities of Central South University, No. 208201025, and National Natural Science Foundation of China, Nos. 81970569 and 81773293.

## Conflict of Interest

The authors declare that the research was conducted in the absence of any commercial or financial relationships that could be construed as a potential conflict of interest.

## Publisher’s Note

All claims expressed in this article are solely those of the authors and do not necessarily represent those of their affiliated organizations, or those of the publisher, the editors and the reviewers. Any product that may be evaluated in this article, or claim that may be made by its manufacturer, is not guaranteed or endorsed by the publisher.
