# Supplementary material for: A Novel Ferroptosis-Related 4-Gene Prognostic Signature for Cholangiocarcinoma and Photodynamic Therapy
Source: Front Oncol. 2021 Oct 12;11:747445. doi: 10.3389/fonc.2021.747445 (PMC8545875; doi:10.3389/fonc.2021.747445)
Supplement: Supplementary file 1 [file DataSheet_1.docx]

Figure S1. The relationship between different WGCNA modules and clinical characteristics. (A-C) The correlation scatter plots of OS, alcoholism, gender and modules, respectively.


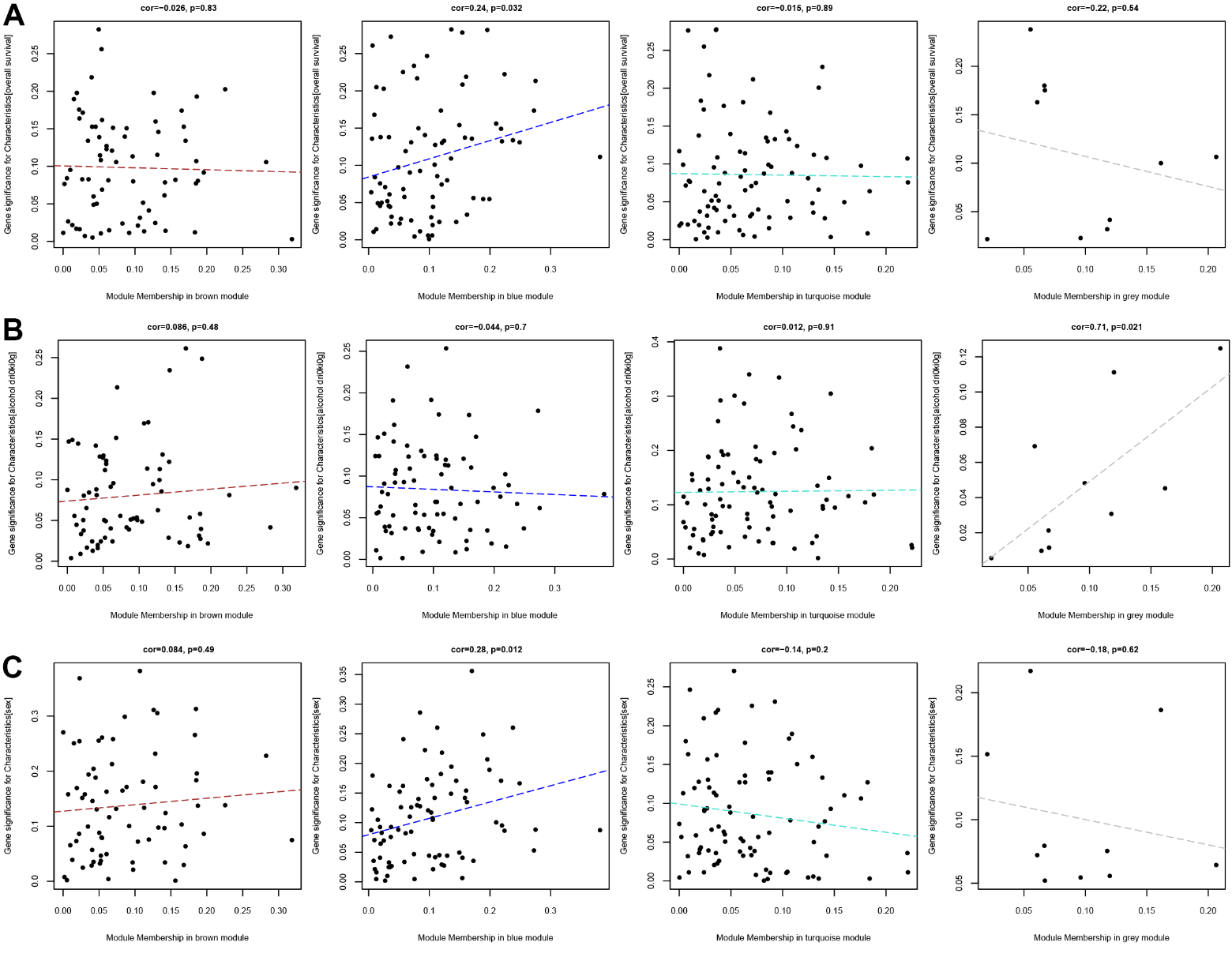


Figure S2. ROC curves of different prognostic models of cholangiocarcinoma. (A) ROC curve for this study. (B) ROC of the CCA prognostic model in the study of Zhang et al. (C) ROC of the CCA prognostic model in the study of Zuo et al.


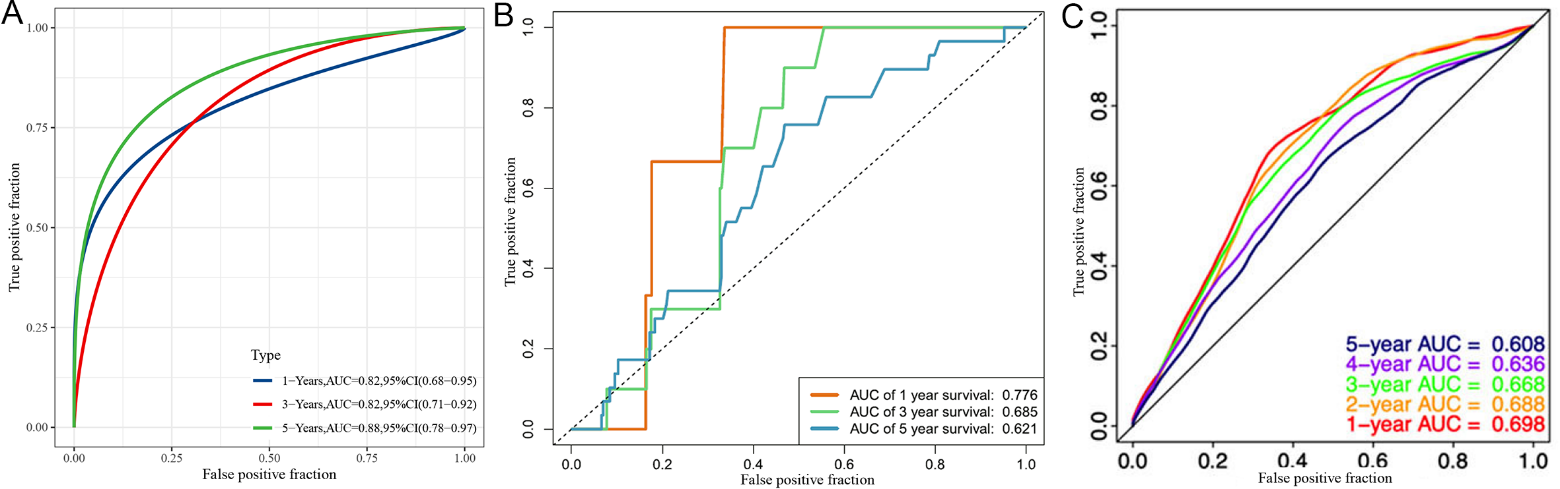


Table S1 The WGCNA module of ferroptosis-related genes and the correlation of clinical features

| Gene | Module | cor_R | Sex | Vascular invasion | Necrosis detected | Diagnosic simptoms | Alcohol drinking | Infection | Cirrhosis of liver | Overall survival | Event death |
| --- | --- | --- | --- | --- | --- | --- | --- | --- | --- | --- | --- |
| ABCC1 | brown | 0.027485 | -0.02402 | 0.079617 | -0.1102 | -0.06567 | 0.016589 | 0.032316 | -0.03923 | -0.17092 | -0.04322 |
| ACO1 | blue | -0.03716 | -0.09231 | -0.02707 | 0.092545 | 0.04039 | -0.10249 | -0.07713 | -0.16248 | 0.021396 | -0.08794 |
| ACSF2 | brown | -0.1111 | -0.18002 | -0.12994 | -0.10414 | -0.00448 | -0.11369 | 0.027298 | -0.00224 | 0.051414 | -0.1064 |
| ACSL1 | brown | -0.01887 | -0.16916 | -0.02728 | -0.07986 | 0.150034 | 0.033221 | -0.02487 | 0.052808 | 0.197413 | -0.30214 |
| ACSL3 | blue | 0.061171 | 0.082 | 0.051271 | 0.05883 | -0.03566 | -0.10887 | -0.05674 | 0.138075 | -0.11886 | 0.041461 |
| ACSL4 | turquoise | 0.220811 | 0.035563 | -0.0019 | 0.010821 | -0.199 | 0.025257 | -0.13635 | 0.095159 | -0.10693 | -0.12186 |
| ACSL5 | brown | -0.05422 | -0.07789 | -0.03877 | 0.041672 | 0.069643 | 0.122878 | 0.362666 | 0.126167 | 0.068415 | -0.08559 |
| ACVR1B | turquoise | 0.138537 | 0.132545 | -0.08488 | -0.07397 | -0.1499 | 0.109196 | 0.140902 | 0.255757 | -0.22812 | 0.014544 |
| AGPAT3 | turquoise | -0.07032 | -0.22543 | 0.061978 | -0.09318 | 0.046536 | -0.18389 | -0.08396 | -0.16098 | 0.033845 | -0.06987 |
| AIFM2 | turquoise | 0.036063 | -0.16186 | -0.05915 | -0.04826 | -0.14445 | -0.04974 | 0.057853 | -0.01722 | -0.03939 | -0.12151 |
| AKR1C1 | blue | -0.09627 | -0.17346 | 0.074793 | -0.09103 | 0.204092 | -0.19113 | -0.08157 | -0.33043 | 0.246804 | -0.14611 |
| AKR1C3 | blue | -0.12078 | -0.21827 | 0.01815 | -0.10791 | 0.094411 | -0.25316 | 0.006636 | -0.29251 | 0.129983 | -0.13082 |
| ALB | brown | 0.040255 | -0.05626 | -0.11747 | -0.00372 | -0.0709 | -0.08826 | 0.054924 | 0.00241 | 0.15246 | -0.12022 |
| ALOX12 | turquoise | -0.02714 | 0.156 | 0.007241 | -0.04454 | 0.02812 | -0.08229 | -0.06882 | -0.12699 | -0.00255 | -0.09438 |
| ALOX12B | grey | -0.02018 | -0.15129 | 0.046829 | -0.04061 | 0.009976 | 0.005557 | -0.00457 | -0.05981 | 0.021427 | 0.045642 |
| ALOX15 | blue | 0.015087 | 0.104878 | 0.035562 | -0.15201 | 0.088181 | 0.080944 | -0.18528 | -0.01525 | -0.04902 | 0.087001 |
| ALOX15B | blue | 0.049069 | -0.088 | 0.053651 | 0.133623 | -0.04927 | -0.00139 | -0.02886 | 0.066585 | 0.097018 | -0.12042 |
| ALOX5 | turquoise | 0.061788 | -0.05135 | -0.03199 | 0.126185 | -0.20543 | -0.08253 | -0.17956 | -0.00987 | -0.18115 | 0.039912 |
| ALOXE3 | brown | 0.040809 | -0.14512 | 0.007114 | 0.07253 | 0.020922 | -0.08128 | -0.05635 | -0.19946 | -0.00518 | -0.00401 |
| ANGPTL7 | brown | -0.0464 | 0.130705 | -0.10397 | -0.13689 | 0.132394 | 0.024312 | 0.07724 | 0.029353 | 0.049585 | 0.178885 |
| ANO6 | brown | 0.12839 | -0.23135 | 0.010519 | -0.02585 | -0.19358 | -0.09929 | -0.02415 | -0.02177 | 0.024506 | -0.242 |
| ARNTL | turquoise | 0.044449 | -0.05053 | 0.085789 | 0.070698 | 0.068039 | 0.096461 | -0.18486 | 0.025783 | -0.02469 | -0.14416 |
| ARRDC3 | turquoise | 0.085591 | -0.00197 | -0.13267 | -0.11946 | 0.054286 | 0.096281 | 0.115649 | 0.152899 | 0.129702 | -0.37211 |
| ASNS | blue | -0.12021 | 0.029426 | -0.06599 | -0.00591 | -0.18698 | 0.119442 | 0.22595 | 0.07174 | 0.073822 | -0.03793 |
| ATF3 | turquoise | 0.028879 | 0.119816 | 0.019606 | -0.00543 | 0.020604 | 0.059417 | 0.034258 | 0.080046 | 0.217063 | -0.22177 |
| ATF4 | brown | 0.022241 | -0.08618 | 0.096152 | -0.19637 | -0.28428 | 0.050066 | 0.01214 | 0.165761 | -0.17514 | 0.072485 |
| ATG13 | blue | 0.195348 | -0.20664 | 0.006537 | 0.114472 | -0.12045 | -0.01876 | -0.0006 | 0.039469 | -0.28186 | 0.03807 |
| ATG16L1 | blue | 0.275022 | -0.08767 | 0.100497 | 0.129471 | -0.09222 | -0.0616 | 0.17883 | 0.036735 | -0.21285 | 0.215463 |
| ATG3 | turquoise | 0.010308 | 0.246352 | -0.00486 | 0.197081 | 0.096495 | 0.055039 | 0.034249 | 0.080379 | 0.076331 | -0.16729 |
| ATG4D | blue | 0.154468 | 0.005962 | -0.03612 | 0.024922 | -0.05986 | -0.02224 | 0.113708 | -0.09663 | 0.278238 | -0.12067 |
| ATG5 | turquoise | 0.076441 | 0.056407 | -0.09411 | 0.070726 | -0.09449 | 0.126992 | 0.26776 | 0.069339 | 0.039574 | -0.15646 |
| ATG7 | turquoise | -0.0596 | 0.054391 | 0.146991 | 0.040204 | -0.00442 | 0.041254 | 0.153601 | 0.029376 | -0.08321 | 0.135055 |
| ATM | turquoise | 0.052808 | 0.270142 | -0.04454 | 0.201948 | 0.045737 | 0.141743 | -0.0648 | 0.102866 | 0.031302 | 0.037807 |
| ATP6V1G2 | blue | 0.136045 | 0.193883 | -0.11352 | -0.01609 | 0.076065 | 0.00808 | -0.08166 | -0.00507 | 0.282123 | 0.023773 |
| AURKA | blue | 0.172538 | 0.035452 | 0.227042 | 0.078129 | -0.09414 | 0.069077 | 0.268308 | 0.189521 | -0.05594 | -0.0187 |
| BAP1 | brown | 0.140947 | -0.09657 | 0.077857 | 0.008731 | -0.12074 | 0.028637 | 0.088134 | -0.01202 | -0.06079 | -0.23512 |
| BECN1 | blue | 0.219562 | -0.09454 | 0.047803 | 0.043723 | -0.22622 | -0.01541 | 0.307262 | 0.079225 | -0.1322 | -0.04572 |
| BID | turquoise | 0.086679 | -0.13116 | -0.06865 | 0.039941 | -0.24138 | -0.07774 | 0.051737 | 0.016406 | -0.01527 | -0.09981 |
| BNIP3 | brown | 0.012935 | -0.03886 | -0.07338 | 0.112384 | -0.22666 | -0.04451 | -0.01067 | 0.129164 | 0.021642 | -0.28012 |
| BRD4 | brown | 0.106955 | -0.38152 | 0.032328 | -0.01389 | 0.069093 | -0.16905 | 0.023424 | 0.117076 | -0.03093 | -0.10996 |
| CA9 | brown | 0.091935 | 0.099921 | -0.07567 | 0.109289 | -0.07401 | 0.05244 | -0.00309 | 0.059998 | -0.01091 | -0.03543 |
| CAPG | turquoise | -0.00184 | -0.05653 | 0.107544 | 0.14187 | -0.07683 | 0.058434 | 0.155357 | 0.160641 | -0.02124 | -0.02451 |
| CAV1 | brown | 0.195851 | -0.08631 | 0.025358 | 0.192251 | -0.09159 | 0.021837 | -0.11366 | -0.09399 | -0.09164 | -0.07304 |
| CBS | brown | -0.10391 | -0.07159 | 0.017455 | -0.0447 | 0.069457 | -0.0487 | 0.039385 | -0.10578 | 0.020954 | -0.01174 |
| CD44 | turquoise | -0.07416 | -0.00724 | -0.02614 | 0.165134 | -0.01409 | 0.179687 | 0.208872 | 0.049387 | -0.07489 | -0.06534 |
| CDKN1A | brown | 0.167886 | 0.028731 | -0.00482 | -0.02341 | 0.051354 | 0.018154 | -0.0896 | -0.04129 | 0.152783 | -0.10679 |
| CDKN2A | blue | 0.103029 | 0.116558 | -0.07851 | 0.097176 | -0.11556 | 0.06918 | 0.074483 | -0.11026 | 0.030413 | 0.062606 |
| CDO1 | brown | -0.18612 | -0.19538 | 0.004897 | -0.02921 | 0.167508 | -0.03922 | 0.137626 | -0.0022 | 0.192674 | -0.15251 |
| CEBPG | blue | -0.11895 | -0.18199 | -0.11547 | -0.0868 | -0.11658 | -0.11321 | 0.065286 | 0.077439 | -0.17321 | 0.028632 |
| CHAC1 | blue | 0.031103 | 0.009418 | 0.113321 | -0.10469 | -0.14127 | 0.031108 | 0.129137 | -0.06878 | -0.0515 | -0.00756 |
| CHMP5 | brown | 0.010005 | 0.064761 | 0.022138 | 0.030263 | -0.13594 | 0.055738 | 0.009172 | -0.01827 | -0.09477 | -0.10215 |
| CHMP6 | grey | 0.060787 | -0.07207 | -0.14647 | 0.13021 | -0.17552 | -0.00956 | -0.0399 | 0.04668 | 0.162591 | -0.25315 |
| CISD1 | blue | -0.01243 | -0.00415 | -0.03677 | 0.008631 | -0.0377 | -0.00149 | -0.09077 | -0.09176 | 0.013844 | 0.081776 |
| CISD2 | turquoise | 0.221319 | -0.01067 | 0.010518 | 0.134861 | -0.18908 | 0.021002 | -0.07679 | 0.022999 | -0.07517 | -0.22273 |
| CP | brown | 0.018114 | -0.07262 | 0.028037 | 0.140955 | -0.11184 | -0.00875 | 0.163106 | 0.02921 | -0.0169 | -0.18648 |
| CS | turquoise | 0.019051 | -0.03598 | 0.026826 | -0.07055 | -0.01454 | 0.034421 | 0.042565 | -0.03785 | -0.01938 | -0.12988 |
| CXCL2 | blue | -0.03666 | 0.075294 | 0.019608 | 0.11459 | -0.04828 | -0.09197 | -0.10123 | -0.18137 | 0.272178 | -0.072 |
| CYBB | turquoise | 0.038529 | 0.025508 | 0.059051 | 0.244902 | -0.03719 | 0.191298 | 0.006943 | 0.092641 | 0.073806 | -0.15279 |
| DDIT3 | turquoise | 0.028191 | -0.03886 | -0.23219 | -0.16498 | -0.1007 | -0.04947 | 0.093636 | 0.155825 | -0.01564 | -0.15023 |
| DDIT4 | blue | 0.104681 | 0.163529 | 0.004335 | 0.088685 | 0.139566 | 0.124586 | 0.140322 | 0.154352 | 0.005532 | -0.06092 |
| DNAJB6 | blue | 0.082117 | 0.139343 | 0.085952 | 0.113377 | 0.031255 | 0.008877 | 0.12028 | 0.035832 | 0.149771 | -0.07924 |
| DPP4 | brown | -0.06418 | -0.11639 | -0.10709 | 0.184174 | -0.08173 | 0.095797 | 0.261252 | 0.065165 | -0.01435 | -0.12992 |
| DRD4 | blue | 0.188832 | 0.248291 | -0.13105 | -0.0574 | -0.11631 | 0.034264 | -0.11884 | -0.01025 | 0.054707 | -0.07374 |
| DRD5 | turquoise | 0.037432 | -0.22008 | -0.01452 | -0.09413 | -0.05504 | -0.13725 | -0.14475 | 0.018845 | -0.00962 | -0.12309 |
| DUOX1 | turquoise | 0.023968 | -0.01085 | -0.08676 | -0.01275 | -0.151 | -0.18812 | -0.1101 | -0.16819 | -0.25458 | 0.130586 |
| DUOX2 | blue | -0.00668 | -0.17903 | -0.0072 | 0.082805 | -0.20585 | -0.05545 | -0.06985 | -0.02261 | -0.26034 | 0.01484 |
| DUSP1 | turquoise | 0.020902 | 0.043039 | -0.01077 | 0.130511 | 0.011859 | 0.099886 | -0.16206 | 0.073772 | 0.183436 | -0.15784 |
| EGFR | turquoise | 0.086335 | -0.13978 | 0.015529 | 0.224638 | -0.06966 | -0.02931 | 0.149441 | -0.14905 | 0.098725 | -0.13905 |
| EGLN2 | turquoise | -0.07285 | -0.03854 | -0.04923 | -0.21302 | -0.119 | -0.03226 | -0.04551 | 0.063514 | 0.003704 | 0.102679 |
| EIF2AK4 | grey | 0.055372 | -0.21691 | -0.16042 | 0.180302 | -0.03082 | -0.06899 | -0.0094 | -0.07007 | 0.237823 | -0.22483 |
| EIF2S1 | turquoise | 0.063645 | -0.13565 | 0.000908 | 0.130897 | -0.03919 | 0.339796 | 0.209509 | 0.078299 | -0.11408 | 0.003381 |
| ELAVL1 | brown | 0.185455 | -0.18351 | 0.05621 | -0.04452 | -0.04757 | 0.027279 | 0.029462 | 0.147553 | -0.10656 | -0.08254 |
| EMC2 | turquoise | -0.13486 | 0.003133 | -0.13818 | -0.12538 | -0.094 | -0.09277 | -0.04778 | -0.12362 | -0.2006 | 0.059772 |
| ENPP2 | turquoise | -0.06866 | 0.042528 | -0.03768 | 0.027867 | -0.14561 | 0.130626 | -0.06378 | 0.081044 | 0.030931 | -0.07434 |
| FADS2 | brown | -0.1327 | -0.09745 | 0.179914 | -0.02377 | 0.066205 | 0.130549 | 0.176542 | -0.06206 | -0.14558 | 0.128839 |
| FANCD2 | blue | 0.075613 | -0.00418 | 0.133193 | 0.105097 | -0.07037 | 0.034944 | 0.380569 | 0.114839 | 0.004298 | 0.123495 |
| FBXW7 | turquoise | 0.06982 | 0.033209 | -0.07702 | -0.05358 | -0.2231 | 0.206955 | 0.048778 | 0.14113 | -0.07075 | 0.057412 |
| FH | blue | -0.03445 | -0.16133 | 0.024088 | 0.004714 | 0.178434 | -0.14131 | -0.14038 | -0.04975 | 0.060954 | -0.11669 |
| FLT3 | blue | -0.03798 | 0.026012 | 0.132687 | 0.015647 | 0.238857 | 0.107022 | -0.16151 | -0.06024 | 0.030935 | 0.152482 |
| FTH1 | turquoise | 0.063717 | -0.12669 | 0.109265 | 0.094447 | -0.00581 | 0.05824 | 0.109504 | 0.107827 | -0.06546 | -0.20979 |
| FTL | turquoise | 0.006327 | -0.17948 | 0.008169 | 0.083648 | 0.044481 | 0.020879 | 0.257883 | 0.132867 | 0.071533 | -0.27825 |
| FTMT | blue | 0.108934 | 0.141845 | 0.025634 | 0.107173 | 0.000833 | 0.173761 | 0.218661 | 0.059654 | 0.100724 | -0.07157 |
| G6PD | turquoise | 0.009189 | -0.01108 | 0.104641 | -0.01768 | -0.1099 | 0.043985 | 0.091721 | -0.03585 | -0.07722 | -0.08869 |
| GABARAPL1 | blue | -0.11656 | 0.044442 | -0.08413 | 0.013601 | 0.06512 | 0.0543 | -0.09272 | -0.02228 | -0.02366 | -0.10038 |
| GABARAPL2 | turquoise | 0.124632 | 0.004287 | 0.025401 | 0.041814 | -0.10209 | -0.02979 | -0.06757 | -0.09555 | -0.08088 | -0.0245 |
| GABPB1 | turquoise | 0.104695 | 0.011289 | -0.24737 | 0.227175 | -0.05808 | 0.266699 | 0.093123 | 0.317721 | 0.050398 | -0.18398 |
| GCH1 | brown | -0.09667 | -0.03341 | 0.048803 | 0.059947 | 0.130159 | 0.053309 | 0.109905 | 0.014242 | 0.112917 | -0.15061 |
| GCLC | brown | -0.16489 | -0.10242 | -0.03111 | -0.02681 | 0.023064 | -0.261 | -0.01608 | -0.13346 | 0.174235 | -0.2855 |
| GCLM | blue | -0.06778 | -0.10976 | 0.019207 | 0.055485 | -0.13719 | -0.09433 | -0.05116 | -0.23131 | 0.089879 | -0.26393 |
| GDF15 | brown | 0.119224 | 0.075846 | 0.01176 | -0.04704 | -0.02761 | -0.09426 | 0.028055 | 0.024055 | 0.040803 | -0.003 |
| GLS2 | brown | -0.12859 | -0.17108 | -0.16672 | -0.15131 | 0.177157 | -0.11303 | 0.038959 | -0.08146 | 0.159414 | -0.0761 |
| GOT1 | blue | 0.004324 | -0.08731 | -0.04826 | -0.02032 | 0.011555 | -0.12378 | -0.07594 | -0.16029 | 0.063377 | 0.038243 |
| GPT2 | brown | -0.08846 | -0.17123 | -0.15138 | -0.02942 | -0.05374 | 0.05097 | 0.256711 | 0.063261 | 0.150654 | -0.15137 |
| GPX2 | blue | -0.09992 | -0.04361 | -0.03776 | -0.10387 | -0.02607 | -0.03366 | 0.169714 | -0.0333 | -0.00075 | -0.14897 |
| GPX4 | brown | 0.183391 | -0.2651 | -0.12931 | 0.135171 | -0.00754 | 0.031358 | -0.04653 | 0.032684 | -0.01193 | -0.10324 |
| GSS | blue | 0.135786 | -0.14853 | -0.0878 | 0.013436 | -0.18331 | -0.04866 | 0.232576 | 0.059069 | -0.10882 | -0.0666 |
| HAMP | brown | -0.0227 | -0.25415 | -0.02049 | 0.036998 | 0.052244 | -0.03736 | 0.159676 | -0.03427 | 0.163926 | -0.09589 |
| HELLS | blue | 0.027115 | -0.00179 | 0.207141 | 0.093945 | -0.08614 | 0.039671 | 0.236732 | 0.098243 | 0.070383 | 0.141713 |
| HERPUD1 | turquoise | -0.016 | -0.0584 | 0.052813 | -0.08631 | 0.155056 | 0.126109 | -0.1165 | 0.089898 | -0.00061 | -0.21865 |
| HIC1 | turquoise | 0.159642 | 0.110132 | -0.03395 | 0.007112 | -0.1034 | 0.115673 | -0.1738 | 0.185532 | -0.04907 | 0.125002 |
| HIF1A | brown | 0.054357 | 0.26042 | 0.028733 | 0.074874 | -0.09624 | 0.119423 | -0.13885 | 0.070475 | -0.16157 | 0.026733 |
| HILPDA | blue | 0.084817 | 0.127465 | -0.04134 | 0.055347 | -0.02326 | 0.123847 | 0.266388 | 0.130308 | 0.092448 | -0.25969 |
| HMGB1 | turquoise | -0.10365 | 0.010341 | -0.00828 | 0.034207 | 0.146638 | 0.088938 | 0.241345 | 0.132202 | 0.142993 | -0.11469 |
| HMOX1 | turquoise | 0.071239 | -0.08255 | 0.064607 | 0.08802 | -0.08203 | 0.121943 | 0.104765 | 0.062366 | 0.211375 | -0.36856 |
| HNF4A | brown | -0.0693 | -0.25744 | -0.05249 | -0.05485 | 0.017782 | -0.21341 | 0.209268 | -0.10443 | 0.151269 | -0.12181 |
| HRAS | brown | -0.00524 | -0.00173 | -0.074 | 0.049677 | -0.09048 | -0.0037 | -0.13028 | 0.027657 | 0.083874 | -0.00458 |
| HSD17B11 | blue | 0.109664 | 0.041703 | -0.11299 | 0.091875 | -0.14256 | 0.02077 | 0.08636 | -0.02871 | 0.127434 | 0.127494 |
| HSF1 | blue | 0.21028 | -0.10039 | 0.048644 | 0.02639 | -0.09629 | -0.07522 | 0.127852 | 0.027493 | -0.15551 | 0.038403 |
| HSPA5 | blue | 0.074856 | 0.046347 | 0.126865 | 0.115249 | -0.28969 | -0.03682 | 0.115582 | 0.102343 | -0.23351 | -0.20294 |
| HSPB1 | blue | 0.020007 | 0.064099 | 0.12073 | 0.003122 | -0.09268 | 0.03912 | 0.220905 | 0.017017 | 0.137627 | -0.19366 |
| IDH1 | brown | -0.18761 | -0.13675 | -0.01491 | -0.11084 | 0.029303 | -0.24818 | 0.026181 | -0.04956 | 0.080439 | -0.0907 |
| IFNG | turquoise | -0.03527 | 0.216869 | -0.12731 | 0.120277 | -0.0575 | 0.387524 | -0.11679 | 0.310681 | 0.05728 | 0.081043 |
| IL33 | turquoise | 0.043198 | 0.063122 | -0.04934 | 0.13317 | 0.154535 | 0.192622 | 0.089935 | 0.095436 | 0.176578 | -0.05164 |
| IL6 | turquoise | 0.113883 | 0.150564 | -0.01448 | -0.01562 | 0.086589 | 0.236838 | 0.023414 | 0.121106 | 0.12359 | -0.16036 |
| IREB2 | turquoise | 0.063459 | -0.17789 | -0.04832 | 0.088466 | -0.03981 | -0.1504 | -0.13747 | -0.17877 | -0.09136 | -0.0157 |
| ISCU | blue | -0.23772 | -0.26041 | -0.18827 | -0.08574 | 0.134621 | -0.0663 | -0.14335 | -0.02573 | 0.133201 | 0.00412 |
| JDP2 | turquoise | -0.14248 | -0.03208 | -0.05428 | -0.08199 | 0.126251 | 0.304482 | -0.06819 | 0.13651 | 0.107781 | -0.12288 |
| JUN | turquoise | -0.03576 | 0.110059 | -0.02824 | -0.16572 | 0.058592 | 0.292056 | 0.157267 | 0.135815 | 0.277214 | -0.16396 |
| KEAP1 | blue | -0.09293 | -0.22233 | 0.031274 | -0.08697 | -0.1465 | -0.03753 | -0.08407 | 0.013386 | -0.14061 | -0.03114 |
| KLHL24 | blue | -0.16195 | 0.134391 | -0.15835 | -0.06451 | 0.031859 | -0.11011 | -0.20527 | 0.037324 | 0.033923 | -0.22616 |
| KRAS | brown | 0.068192 | 0.2126 | 0.024393 | 0.168132 | -0.20324 | -0.15155 | 0.114108 | 0.03896 | -0.12033 | 0.013669 |
| LAMP2 | blue | -0.02165 | -0.08292 | -0.1226 | -0.05056 | 0.009069 | -0.034 | -0.22973 | -0.08428 | 0.049943 | -0.07033 |
| LINC00336 | grey | -0.11981 | -0.0554 | -0.00359 | 0.005365 | -0.04012 | 0.111087 | -0.10858 | -0.02748 | 0.041284 | 0.001143 |
| LINC00472 | turquoise | -0.04407 | 0.059889 | 0.006361 | -0.0715 | -0.02217 | 0.022037 | -0.09509 | -0.07573 | -0.08805 | 0.04345 |
| LONP1 | blue | 0.019054 | -0.04077 | -0.06278 | -0.03552 | -0.19445 | 0.150788 | 0.098114 | 0.198523 | 0.075187 | -0.28836 |
| LPCAT3 | brown | -0.18497 | -0.31288 | 0.070982 | -0.06002 | 0.014578 | -0.05801 | 0.191426 | 0.049163 | -0.07676 | -0.1535 |
| LPIN1 | brown | -0.03545 | -0.19337 | 0.048056 | -0.03979 | -0.03673 | 0.012528 | -0.00797 | 0.00443 | 0.0827 | -0.15113 |
| LURAP1L | blue | -0.07006 | -0.08432 | -0.15596 | 0.033966 | -0.03598 | -0.05605 | -0.12779 | -0.22002 | 0.025869 | 0.1895 |
| MAFG | blue | 0.272307 | -0.0524 | 0.121052 | 0.026862 | -0.03168 | -0.17801 | -0.00755 | -0.09433 | -0.17344 | -0.03737 |
| MAP1LC3A | brown | 0.042316 | 0.034688 | 0.098174 | -0.11355 | 0.109966 | -0.01969 | 0.230874 | 0.004777 | -0.05987 | 0.025003 |
| MAP1LC3B | turquoise | 0.134589 | -0.07018 | -0.00391 | 0.147524 | -0.08559 | 0.097255 | 0.081155 | 0.186447 | 0.065525 | -0.23208 |
| MAP1LC3C | blue | 0.084624 | 0.285714 | 0.134561 | 0.009283 | -0.07101 | 0.053329 | 0.065316 | -0.04768 | -0.01158 | -0.078 |
| MAP3K5 | turquoise | 0.024103 | -0.0898 | 0.034499 | -0.08263 | 0.040169 | 0.129014 | 0.129362 | 0.09258 | -0.00973 | -0.02673 |
| MAPK1 | brown | 0.015121 | -0.24999 | 0.029652 | 0.018554 | -0.08316 | -0.14394 | 0.028322 | 0.029726 | -0.18944 | 0.031213 |
| MAPK14 | turquoise | 0.184177 | -0.00281 | -0.13248 | -0.03401 | -0.02289 | -0.11848 | -0.07584 | 0.042691 | -0.06354 | 0.042827 |
| MAPK3 | blue | 0.008257 | -0.03483 | 0.010294 | 0.054933 | -0.10943 | 0.146411 | 0.454425 | 0.086847 | -0.01075 | -0.00334 |
| MAPK8 | brown | -0.04524 | -0.18775 | -0.10589 | -0.03831 | 0.043663 | -0.12801 | -0.05601 | -0.04873 | 0.152427 | 0.032998 |
| MAPK9 | brown | -0.00189 | 0.00736 | 0.038573 | -0.12372 | 0.146534 | -0.14702 | -0.15999 | -0.11123 | 0.076002 | 0.169467 |
| MIOX | grey | -0.16144 | -0.18625 | 0.059859 | -0.08449 | 0.045836 | 0.045042 | -0.17278 | 0.105163 | -0.09991 | 0.098794 |
| MIR212 | turquoise | 0.081833 | 0.000152 | 0.011447 | -0.01939 | -0.10647 | 0.054852 | -0.07934 | 0.085986 | -0.08686 | 0.189535 |
| MIR30B | turquoise | -0.00369 | 0.113076 | -0.12208 | -0.06784 | -0.09144 | -0.10265 | -0.15677 | -0.11263 | -0.09864 | 0.148059 |
| MIR4715 | blue | 0.144397 | 0.170586 | 0.102423 | 0.001228 | 0.076464 | -0.06666 | -0.10717 | -0.23188 | 0.024032 | 0.052837 |
| MIR9-1 | brown | -0.02284 | 0.368183 | -0.12693 | -0.18717 | 0.000812 | 0.080018 | 0.040393 | 0.023003 | 0.015814 | -0.01954 |
| MT1G | brown | 0.026597 | -0.15103 | 0.033621 | 0.20137 | 0.139235 | 0.064734 | 0.233829 | -0.00583 | 0.082776 | -0.09661 |
| MT3 | turquoise | -0.02375 | 0.209053 | 0.07607 | 0.151054 | 0.089548 | 0.146063 | 0.170085 | 0.00688 | 0.171727 | -0.19147 |
| MTDH | blue | 0.056754 | -0.16193 | -0.06674 | -0.19681 | -0.13336 | -0.13638 | 0.017125 | 0.015284 | -0.2251 | -0.05874 |
| MTOR | brown | -0.08598 | -0.29879 | 0.056854 | -0.0133 | -0.01428 | 0.03882 | 0.063117 | 0.020954 | -0.13912 | -0.0807 |
| MUC1 | blue | -0.0336 | -0.03261 | 0.192017 | 0.063567 | -0.12395 | 0.05273 | 0.086128 | 0.148892 | -0.13789 | -0.06743 |
| MYB | blue | -0.12402 | 0.026864 | 0.113756 | -0.04417 | -0.01521 | 0.112672 | 0.425129 | 0.098449 | 0.132632 | -0.05154 |
| NCF2 | turquoise | 0.03381 | -0.02009 | -0.00206 | 0.128215 | -0.13058 | 0.169408 | -0.08421 | 0.064426 | -0.04197 | -0.00722 |
| NCOA4 | blue | -0.05123 | -0.15166 | 0.079173 | 0.035755 | 0.183692 | -0.09252 | -0.2039 | -0.1892 | 0.021817 | -0.11298 |
| NF2 | brown | 0.31852 | -0.0749 | -0.08944 | 0.113111 | -0.17972 | 0.089855 | -0.22009 | 0.165971 | -0.00281 | 0.047538 |
| NFE2L2 | turquoise | 0.024298 | 0.091103 | 0.085249 | 0.024887 | 0.106797 | -0.18707 | 0.08606 | -0.04853 | 0.063696 | 0.056527 |
| NFS1 | turquoise | -0.06184 | -0.03265 | -0.18834 | -0.0123 | 0.011708 | -0.07338 | 0.068827 | 0.003422 | 0.006192 | -0.05547 |
| NGB | blue | 0.381233 | 0.087346 | 0.140651 | -0.01852 | 0.028914 | -0.07801 | 0.06514 | -0.08741 | -0.11139 | 0.150845 |
| NNMT | turquoise | 0.014571 | 0.119276 | -0.00182 | 0.137823 | 0.107107 | 0.009853 | 0.028298 | -0.14315 | 0.024345 | -0.14175 |
| NOS2 | blue | 0.098059 | 0.120683 | 0.071795 | -0.04417 | -0.00765 | 0.029734 | -0.12226 | 0.014488 | -0.00543 | 0.029013 |
| NOX1 | blue | -0.15454 | 0.040787 | 0.091492 | -0.08371 | -0.01312 | -0.01181 | 0.280422 | -0.02114 | 0.208144 | -0.26179 |
| NOX3 | blue | 0.113201 | 0.260246 | 0.158394 | 0.03319 | 0.034229 | 0.101769 | -0.04766 | 0.008802 | 0.085059 | 0.047969 |
| NOX4 | turquoise | 0.008381 | -0.03177 | -0.0006 | -0.10745 | -0.04791 | 0.156249 | 0.015306 | 0.12881 | 0.019576 | -0.13927 |
| NQO1 | blue | 0.01281 | -0.01558 | 0.117599 | -0.05897 | -0.00105 | 0.063147 | 0.195185 | 0.14882 | -0.20502 | 0.130849 |
| NRAS | brown | 0.142538 | 0.123399 | 0.19729 | 0.195322 | -0.0131 | 0.234345 | 0.115094 | -0.07824 | 0.01406 | 0.003177 |
| OTUB1 | brown | 0.063045 | -0.00392 | -1.31E-05 | 0.13508 | -0.28696 | 0.02393 | 0.083293 | -0.05068 | -0.08142 | 0.092326 |
| OXSR1 | blue | -0.03315 | 0.024875 | 0.034525 | -0.01308 | -0.14319 | 0.190606 | 0.311957 | 0.152296 | -0.04525 | -0.15856 |
| PANX1 | turquoise | 0.175813 | -0.10635 | -0.04801 | 0.094862 | -0.10207 | 0.104522 | -0.01602 | 0.075953 | 0.097469 | -0.25636 |
| PCBP1 | turquoise | 0.086951 | -0.06171 | 0.115444 | 0.055649 | -0.05997 | -0.02972 | -0.03181 | -0.01414 | 0.029667 | 0.05761 |
| PCBP2 | turquoise | -2.19E-06 | -0.07335 | -0.28719 | -0.04729 | -0.13685 | 0.067472 | 0.109263 | 0.146221 | -0.11662 | -0.18826 |
| PCK2 | brown | -0.13105 | -0.30539 | -0.04135 | -0.08684 | 0.102368 | -0.08537 | 0.196899 | -0.02795 | 0.11508 | -0.18795 |
| PEBP1 | blue | -0.17027 | -0.35562 | -0.14669 | -0.16896 | 0.130768 | -0.14695 | 0.009923 | -0.0581 | 0.13536 | -0.18416 |
| PGD | turquoise | -0.00028 | 0.004193 | 0.077056 | 0.040406 | -0.16587 | 0.11461 | 0.233753 | 0.020623 | -0.01821 | -0.14561 |
| PHKG2 | turquoise | -0.03804 | -0.07013 | 0.055118 | 0.015769 | -0.09237 | -0.0971 | 0.228955 | -0.08031 | 0.051396 | 0.089298 |
| PIK3CA | turquoise | -0.08397 | 0.01429 | -0.15045 | -0.03555 | 0.058276 | -0.10445 | -0.18654 | 0.101462 | 0.134186 | -0.19633 |
| PLIN4 | brown | 4.76E-05 | 0.270441 | -0.0418 | 0.037959 | 0.064749 | 0.08717 | -0.02973 | 0.108257 | -0.0108 | -0.13966 |
| PML | turquoise | -0.03339 | -0.11377 | 0.000307 | 0.113781 | 0.02735 | 0.253564 | -0.01051 | 0.126816 | -0.09507 | -0.02721 |
| PRDX1 | turquoise | 0.128778 | -0.15993 | 0.024698 | 0.091322 | -0.19209 | 0.042009 | 0.077821 | -0.03654 | 0.047966 | -0.25912 |
| PRDX6 | blue | -0.19903 | -0.18873 | -0.10266 | -0.04981 | 0.184381 | -0.08602 | -0.11481 | -0.02003 | 0.054369 | -0.15849 |
| PRKAA1 | grey | -0.20639 | 0.064348 | -0.15981 | 0.011032 | -0.02269 | -0.12463 | -0.00346 | 0.029388 | -0.1064 | 0.199415 |
| PRKAA2 | blue | -0.01885 | -0.09236 | -0.12614 | 0.010352 | -0.12493 | -0.09072 | -0.13284 | -0.13894 | 0.045664 | -0.12107 |
| PRNP | turquoise | -0.01883 | -0.12775 | -0.07836 | -0.03278 | 0.042152 | -0.03628 | -0.11596 | 0.037157 | -0.13747 | -0.04396 |
| PROM2 | blue | 0.158558 | -0.14121 | -0.03065 | -0.07233 | 0.010091 | -0.17317 | -0.0092 | -0.09618 | -0.1371 | 0.134475 |
| PSAT1 | brown | -0.22569 | -0.13786 | -0.06024 | -0.04854 | 0.07819 | 0.081168 | 0.201824 | 0.119354 | 0.202423 | -0.16597 |
| PTGS2 | turquoise | -0.05823 | 0.126618 | 0.1858 | -0.0812 | 0.113803 | 0.132234 | 0.076578 | 0.08838 | 0.012332 | 0.013625 |
| RB1 | turquoise | -0.08903 | 0.139283 | 0.05896 | 0.181259 | 0.056843 | 0.119439 | 0.092069 | 0.105205 | 0.096236 | -0.20026 |
| RELA | brown | 0.282488 | -0.22726 | -0.01121 | 0.202348 | -0.22478 | 0.0411 | -0.02918 | -0.02457 | 0.105057 | -0.23661 |
| RGS4 | blue | -0.07009 | -0.12587 | -0.13851 | -0.01361 | -0.13244 | -0.06501 | -0.10166 | -0.0396 | 0.130389 | 0.039817 |
| RIPK1 | turquoise | 0.019895 | 0.040436 | -0.00529 | 0.122998 | -0.04127 | -0.00762 | -0.22763 | 0.139052 | -0.03713 | -0.12547 |
| RPL8 | brown | 0.060436 | 0.04924 | -0.00526 | 0.030033 | -0.14595 | 0.091476 | 0.077875 | 0.011377 | -0.12251 | 0.017246 |
| RRM2 | blue | 0.106169 | -0.02127 | 0.206613 | 0.139822 | -0.06745 | 0.054132 | 0.283962 | 0.10521 | 0.043842 | 0.003709 |
| SAT1 | turquoise | 0.027688 | -0.12994 | 0.141597 | 0.019321 | 0.110129 | 0.072266 | 0.037989 | 0.161076 | 0.044171 | -0.15923 |
| SAT2 | blue | -0.21846 | -0.17028 | -0.1701 | 0.000973 | 0.006759 | -0.10187 | -0.08535 | -0.06607 | 0.149187 | -0.27388 |
| SCD | brown | 0.052524 | -0.04583 | 0.188014 | 0.012234 | 0.023857 | 0.028521 | 0.148528 | -0.03066 | -0.01044 | -0.0987 |
| SCP2 | brown | -0.04949 | -0.25533 | -0.12728 | -0.10907 | 0.126343 | -0.12701 | -0.03333 | -0.09553 | 0.281979 | -0.23 |
| SESN2 | brown | -0.03022 | -0.15774 | 0.050388 | 0.000385 | -0.12404 | 0.084231 | 0.086355 | -0.03493 | -0.00669 | 0.043344 |
| SETD1B | turquoise | -0.0084 | -0.16297 | 0.082633 | -0.21726 | 0.047419 | -0.14563 | -0.15226 | -0.01313 | -0.27601 | 0.177805 |
| SIRT1 | grey | 0.066951 | 0.051867 | -0.09635 | -0.13688 | 0.210958 | 0.011352 | -0.0631 | 0.167209 | 0.175275 | 0.074175 |
| SLC11A2 | grey | -0.06652 | -0.07917 | -0.07262 | -0.19979 | -0.15079 | -0.02118 | -0.09304 | 0.073869 | -0.17972 | -0.03345 |
| SLC1A4 | blue | -0.22405 | -0.08614 | -0.06455 | -0.0758 | -0.20036 | 0.088892 | 0.247755 | 0.098793 | 0.222331 | -0.1297 |
| SLC1A5 | blue | 0.024967 | 0.069736 | 0.01346 | -0.09123 | -0.08119 | 0.078282 | 0.242796 | 0.210162 | -0.20282 | 0.020941 |
| SLC2A1 | blue | 0.149767 | -0.04898 | 0.06931 | 0.025139 | -0.06138 | 0.120735 | 0.208532 | 0.196117 | -0.15368 | -0.05695 |
| SLC2A12 | brown | -0.05133 | 0.031894 | -0.16766 | -0.1305 | 0.036474 | 0.050438 | 0.087827 | -0.12429 | 0.114151 | -0.14711 |
| SLC2A14 | turquoise | 0.092502 | 0.230637 | -0.03223 | 0.036265 | -0.04694 | 0.333854 | 0.042277 | 0.182555 | 0.13218 | -0.30709 |
| SLC2A3 | turquoise | 0.10615 | 0.18314 | -0.05063 | 0.08382 | -0.01659 | 0.243697 | 0.072018 | 0.156671 | 0.132297 | -0.28212 |
| SLC2A6 | blue | 0.160547 | 0.153696 | -0.01861 | 0.169802 | -0.02928 | 0.035381 | 0.008098 | -0.0502 | 0.218853 | -0.03446 |
| SLC2A8 | brown | 0.053485 | -0.07904 | 0.052178 | 0.025516 | 0.092657 | -0.04885 | 0.20454 | -0.01931 | 0.256064 | -0.21848 |
| SLC38A1 | brown | 0.052347 | 0.037001 | 0.086317 | 0.022776 | -0.02046 | -0.11185 | 0.007926 | -0.015 | -0.1081 | 0.22198 |
| SLC39A14 | brown | -0.04222 | -0.20368 | 0.153169 | 0.002126 | -0.0491 | -0.01587 | 0.077512 | -0.09114 | 0.048384 | -0.16621 |
| SLC39A8 | brown | 0.060242 | -0.16226 | 0.044385 | 0.02642 | 0.021347 | 0.040238 | 0.16007 | -0.00221 | 0.12694 | -0.40954 |
| SLC3A2 | blue | -0.00943 | -0.07031 | 0.067895 | -0.2723 | -0.08228 | 0.056234 | 0.185558 | 0.041755 | -0.16755 | 0.098701 |
| SLC40A1 | blue | -0.0351 | -0.02419 | -0.05955 | -0.14474 | 0.057426 | -0.16162 | -0.21496 | -0.04467 | 0.044095 | 0.175661 |
| SLC7A11 | blue | 0.105498 | -0.10487 | -0.08732 | -0.01062 | -0.17436 | 0.068695 | 0.293737 | 0.04555 | 0.057656 | -0.13352 |
| SLC7A5 | blue | 0.005461 | -0.12173 | 0.26947 | -0.19874 | 0.056381 | -0.01065 | 0.171294 | 0.098942 | -0.13543 | -0.06692 |
| SNX4 | blue | -0.0799 | 0.128882 | -0.09258 | -0.0355 | 0.02717 | -0.13022 | -0.22442 | -0.01798 | 0.216889 | -0.13082 |
| SOCS1 | turquoise | 0.129629 | 0.005726 | 0.107552 | 0.14682 | -0.0327 | 0.13454 | -0.01861 | 0.050382 | 0.035307 | -0.07488 |
| SP1 | brown | 0.126046 | -0.31099 | -0.03456 | -0.1553 | -0.12743 | 0.062206 | 0.098014 | 0.179552 | -0.19771 | 0.034103 |
| SQSTM1 | blue | 0.010476 | -0.02089 | 0.113121 | -0.07428 | 0.205293 | -0.12366 | 0.008267 | -0.15186 | -0.08423 | -0.05305 |
| SRC | blue | 0.248664 | -0.16589 | 0.072873 | -0.00529 | -0.12524 | -0.03677 | 0.193341 | 0.004768 | -0.13072 | 0.214238 |
| STAT3 | turquoise | 0.031269 | -0.06571 | -0.08858 | -0.10902 | -0.0906 | 0.078822 | -0.01194 | 0.077351 | -0.05155 | -0.17173 |
| STEAP3 | brown | 0.00692 | -0.15731 | -0.04042 | 0.120502 | 0.118634 | -0.14873 | 0.140846 | -0.02742 | -0.0266 | -0.06249 |
| TAZ | brown | 0.082434 | -0.16406 | 0.121697 | 0.041255 | 0.121206 | 0.041274 | 0.012282 | -0.06675 | 0.023255 | -0.14848 |
| TFAP2C | brown | -0.03472 | -0.09903 | 0.151645 | -0.12183 | 0.001594 | 0.023917 | -0.17468 | 0.028656 | -0.13382 | 0.024783 |
| TFR2 | brown | -0.11259 | -0.13354 | 0.057318 | 0.017731 | 0.11548 | -0.17061 | -0.01871 | -0.13272 | -0.01344 | -0.0342 |
| TFRC | blue | 0.053235 | 0.125842 | 0.052702 | 0.156675 | 0.03565 | -0.03676 | 0.110991 | -0.02756 | 0.027826 | -0.09725 |
| TGFBR1 | turquoise | 0.109005 | 0.189324 | 0.016796 | 0.180762 | -0.14885 | 0.201877 | -0.0317 | 0.139406 | 0.087831 | -0.24331 |
| TLR4 | turquoise | 0.036862 | -0.02237 | 0.025222 | 0.090832 | 0.120611 | 0.198455 | -0.00505 | 0.134225 | 0.108587 | -0.15823 |
| TMBIM4 | grey | -0.09601 | -0.05416 | -0.11511 | -0.05061 | 0.126406 | -0.04815 | -0.10243 | 0.02437 | -0.02242 | 0.044445 |
| TNFAIP3 | turquoise | 0.087895 | 0.009947 | 0.119371 | 0.193199 | -0.05775 | 0.195388 | -0.04093 | 0.133558 | 0.167104 | -0.1074 |
| TP53 | brown | 0.097324 | 0.020678 | 0.005335 | -0.0345 | -0.08479 | 0.050312 | -0.25239 | 0.023964 | -0.07948 | -0.03222 |
| TP63 | blue | 0.105222 | -0.10759 | 0.058715 | -0.14969 | 0.062584 | -0.12071 | -0.02021 | -0.11375 | 0.019437 | -0.03582 |
| TRIB3 | turquoise | -0.02686 | 0.093594 | -0.11616 | -0.00241 | -0.07422 | -0.04598 | -0.00724 | 0.018161 | 0.031213 | 0.005634 |
| TSC22D3 | turquoise | -0.04945 | 0.087704 | 0.028999 | 0.090466 | 0.147903 | 0.300381 | -0.08291 | 0.132631 | 0.139238 | -0.04028 |
| TUBE1 | brown | -0.17047 | -0.06355 | -0.1732 | -0.15393 | 0.171773 | 0.053756 | -0.02163 | 0.012994 | 0.133456 | -0.14274 |
| TXNIP | turquoise | 0.058735 | -0.03788 | -0.007 | 0.152372 | 0.12695 | 0.286044 | 0.13116 | 0.227784 | 0.116002 | -0.07055 |
| TXNRD1 | blue | 0.058995 | -0.03325 | -0.01128 | 0.01972 | -0.13324 | -0.12334 | 0.198869 | -0.11316 | -0.05714 | -0.23976 |
| UBC | blue | 0.129456 | -0.0436 | 0.03346 | 0.037321 | 0.014541 | 0.085768 | 0.11526 | 0.296788 | -0.07973 | -0.03694 |
| ULK1 | turquoise | 0.048452 | -0.09526 | 0.075312 | -0.07151 | -0.0118 | -0.15886 | -0.09284 | 0.015024 | -0.04294 | -0.03376 |
| ULK2 | brown | 0.141637 | -0.03423 | -0.07576 | -0.37472 | 0.041266 | -0.12179 | -0.21067 | 0.077524 | -0.07859 | 0.070463 |
| VDAC2 | turquoise | -0.14645 | 0.092426 | -0.08015 | 0.050947 | -0.00914 | 0.094952 | 0.048936 | -0.01855 | -0.00341 | 0.066526 |
| VDAC3 | brown | -0.07367 | 0.131677 | 0.186546 | -0.02823 | 0.046935 | 0.055321 | -0.1585 | -0.10124 | -0.10572 | -0.06763 |
| VEGFA | turquoise | 0.107338 | 0.077602 | -0.031 | -0.03517 | -0.15477 | 0.018505 | 0.189334 | 0.058904 | -0.02844 | -0.19782 |
| VLDLR | turquoise | -0.1301 | -0.04952 | 0.039769 | -0.28419 | 0.051768 | -0.00154 | -0.18058 | -0.00118 | -0.11154 | -0.05155 |
| WIPI1 | grey | -0.11765 | 0.075136 | -0.22702 | 0.001132 | -0.08647 | 0.03068 | -0.15616 | 0.098143 | -0.03187 | 0.014247 |
| WIPI2 | blue | 0.057193 | -0.24073 | -0.07689 | 0.043174 | -0.08049 | -0.23143 | 0.139068 | -0.04692 | 0.06808 | -0.29357 |
| XBP1 | brown | -0.05009 | -0.08736 | -0.01524 | -0.22151 | -0.02343 | -0.12943 | -0.08999 | 0.055197 | -0.13882 | -0.08858 |
| YWHAE | brown | 0.156105 | 0.000811 | -0.07213 | 0.098922 | -0.12491 | -0.02275 | -0.01257 | 0.066991 | 0.081685 | -0.18621 |
| YY1AP1 | turquoise | -0.18202 | 0.127154 | 0.128733 | 0.026339 | 0.070746 | -0.2034 | -0.11199 | -0.15003 | -0.00842 | 0.06466 |
| ZEB1 | turquoise | 0.140729 | 0.076343 | 0.042069 | 0.009082 | 0.138672 | 0.148789 | -0.14784 | 0.133631 | 0.027984 | -0.08811 |
| ZFP36 | turquoise | -0.03547 | 0.035947 | -0.08455 | 0.115867 | 0.062076 | 0.110006 | 0.040068 | 0.131136 | 0.276547 | -0.157 |
| ZNF419 | brown | 0.039863 | 0.027846 | -0.19511 | 0.00853 | -0.24463 | -0.14155 | -0.1952 | 0.039802 | -0.21841 | 0.113487 |
